# Macrophages facilitate tumor cell PD‐L1 expression via an IL‐1β‐centered loop to attenuate immune checkpoint blockade

**DOI:** 10.1002/mco2.242

**Published:** 2023-03-30

**Authors:** Cheng Xu, Yu Xia, Bai‐Wei Zhang, Emmanuel Kwateng Drokow, Hua‐Yi Li, Sen Xu, Zhen Wang, Si‐Yuan Wang, Ping Jin, Tian Fang, Xiao‐Ming Xiong, Pu Huang, Ning Jin, Jia‐Hong Tan, Qing Zhong, Yu‐Xin Chen, Qi Zhang, Yong Fang, Fei Ye, Qing‐Lei Gao

**Affiliations:** ^1^ Department of Gynecological Oncology Tongji Hospital Tongji Medical College Huazhong University of Science and Technology Wuhan China; ^2^ National Clinical Research Center for Obstetrics and Gynecology Cancer Biology Research Center (Key Laboratory of the Ministry of Education) Tongji Hospital Tongji Medical College Huazhong University of Science and Technology Wuhan China; ^3^ Department of Neurosurgery Tongji Hospital Tongji Medical College Huazhong University of Science and Technology Wuhan China; ^4^ Department of Radiation Oncology Zhengzhou University People's Hospital & Henan Provincial People's Hospital Zhengzhou China; ^5^ Department of Obstetrics and Gynecology The Second Affiliated Hospital Wenzhou Medical University Wenzhou China; ^6^ Department of Obstetrics and Gynecology The First People's Hospital of Yunnan Province The Affiliated Hospital of Kunming University of Science and Technology Kunming China; ^7^ Department of Plastic and Cosmetic Surgery Tongji Hospital Tongji Medical College Huazhong University of Science and Technology Wuhan China

**Keywords:** immune checkpoint blockade, interleukin‐1β, programmed death‐ligand 1, tumor‐immune microenvironment, tumor‐associated macrophages

## Abstract

Tumor‐associated macrophages (TAMs) play critical roles in reprogramming other immune cells and orchestrating antitumor immunity. However, the interplay between TAMs and tumor cells responsible for enhancing immune evasion remains insufficiently understood. Here, we revealed that interleukin (IL)‐1β was among the most abundant cytokines within the in vitro tumor‐macrophage coculture system, and enhanced IL‐1β expression was associated with impaired cytotoxicity of CD8^+^ T cells in human ovarian cancer, indicating the possibility that IL‐1β mediated immunosuppression during tumor‐TAMs crosstalk. Mechanistically, we demonstrated that IL‐1β significantly boosted programmed death‐ligand 1 (PD‐L1) expression in tumor cells via the activation of the nuclear factor‐κb signaling cascade. Specifically, IL‐1β released from TAMs was triggered by lactate, the anaerobic metabolite of tumor cells, in an inflammasome activation‐dependent manner. IL‐1β sustained and intensified immunosuppression by promoting C‐C motif chemokine ligand 2 secretion in tumor cells to fuel TAMs recruitment. Importantly, IL‐1β neutralizing antibody significantly curbed tumor growth and displayed synergistic antitumor efficacies with anti‐PD‐L1 antibody in tumor‐bearing mouse models. Together, this study presents an IL‐1β‐centered immunosuppressive loop between TAMs and tumor cells, highlighting IL‐1β as a candidate therapeutic target to reverse immunosuppression and potentiate immune checkpoint blockade.

## Graphical abstract

The pro‐inflammatory cytokine IL‐1β could be triggered and released from macrophages (Mφ) by lactate derived from tumor cells. Interleukin (IL)‐1β, in turn, drove an immune inhibitory phenotype on tumor cells, especially on the increase in programmed death‐ligand 1, by activating the nuclear factor‐κB (NF‐κB) signaling pathway. Thus, tumor‐released lactate and Mφ‐derived IL‐1β mediated the crosstalk loop and caused immunosuppression. Moreover, activated NF‐κB signaling promoted C‐C motif chemokine ligand 2 secretion in tumor cells, leading to further monocyte recruitment and replenishment of the Mφ population, which maintained the malignant feedback loop (Figure 7).

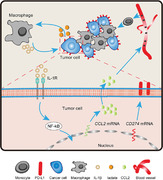

## 1 INTRODUCTION

Immune checkpoint blockade (ICB) has become a spotlight for cancer research and clinical therapies over the past two decades owing to its extensive benefits in counteracting multiple cancers, including melanoma, non‐small cell lung cancer (NSCLC), and others.^1^, ^2^ However, several malignancies such as ovarian cancer and glioma respond poorly to ICB.^3^, ^4^ Even in lung cancer, where ICB works well, the response rate is less than 50%.^1^ The generally poor clinical efficacy associated with the ICB can be attributed to the complex tumor‐immune microenvironment (TIME).

TIME, which is composed of multiple infiltrating immune cells such as tumor‐associated macrophages (TAMs), regulatory T cells (Tregs), natural killer cells, CD8^+^ T lymphocytes, and myeloid‐derived suppressor cells (MDSCs), modulates the efficacy of ICB.^5^, ^6^ Of these, TAMs are critical in shaping immune responses due to their high frequency (up to 50%) via direct and indirect manners.^7^ Immune checkpoint ligands such as programmed death‐ligand 1 (PD‐L1) and B7‐H4 are expressed by TAMs and directly suppress CD8^+^ T lymphocytes.^8^, ^9^ Moreover, multiple molecules, including interleukin (IL)‐10, TGF‐β, and reactive oxygen species, which are secreted by TAMs,^10^ act on CD8^+^ T lymphocytes and inhibit their cytotoxicity.^11^, ^12^, ^13^ TAMs can also indirectly recruit other immunosuppressive cell populations, including Tregs.^14^ Additionally, TAMs prevent T‐cell recruitment by regulating the vascular structure, extracellular matrix (ECM), and chemokine content.^15^ However, the crosstalk between macrophages (Mφ) and tumor cells responsible for reshaping the TIME is still unclear. Therefore, studies of mechanisms underlying TAM–tumor cell communications and their effects on tumor immunity are urgently demanded.

In addition to tumor cells, various infiltrating and resident stromal cells, secreted bioactive factors, and ECM make up a complex tumor micro‐ecosystem within tumor tissues.^16^ Cytokines are the most abundant secreted factors in the TME and mediate cell‐to‐cell communication between stromal cells and tumor cells, either affecting their proliferation and metastasis (IL‐1β, IL‐6, IL‐10, interferon (IFN)‐γ, TGF‐β, and VEGF) or modulating the TIME, reshaping immune responses to tumor cells (IL‐2, IL‐4, IL‐7, IFN‐γ, transforming growth factor‐β (TGF‐β),vascular endothelial growth factor (VEGF), and granulocyte macrophage‐colony stimulating factor (GM‐CSF)).^17^, ^18^ Moreover, diverse metabolites such as lactate, amino acids, glucose, and lipids flooded in the TME have profound impacts on cellular communication, which are required to regulate tumor progression.^19^ For example, increased glucose uptake by tumor cells can blunt the antitumor effect of T cells by inhibiting their glycolysis.^20^ Lactate, a side product of the Warburg effect in tumor cells, promotes the differentiation of Tregs and M2‐like Mφ, deteriorating the immune microenvironment.^21^, ^22^ Besides, emerging evidence shows that amino acids such as glutamine, tryptophan, and methionine function as critical immune accelerators, enhancing antitumor immune responses.^23^ Thus, to further investigate the communication between tumor cells and Mφ, we need to clarify complex components that mediate the crosstalk.

Here, we report that IL‐1β is one of the most abundant cytokines secreted by THP1‐derived Mφ (THP1 Mφ) following direct co‐culturing with tumor cell lines and functions as a critical PD‐L1‐inducer in tumor cells to blunt the cytotoxicity of T cells. Meanwhile, lactate released by tumor cells inversely triggers the secretion of IL‐1β in Mφ. Thus, IL‐1β and lactate mediate the feedback crosstalk between Mφ and tumor cells to weaken antitumor immunity, designing a synergistic antitumor effect when ICB is combined with IL‐1β blockade.

## 2 RESULTS

### 2.1 TAM infiltration and IL‐1β secretion are associated with immunosuppressive TME

**FIGURE 1:**
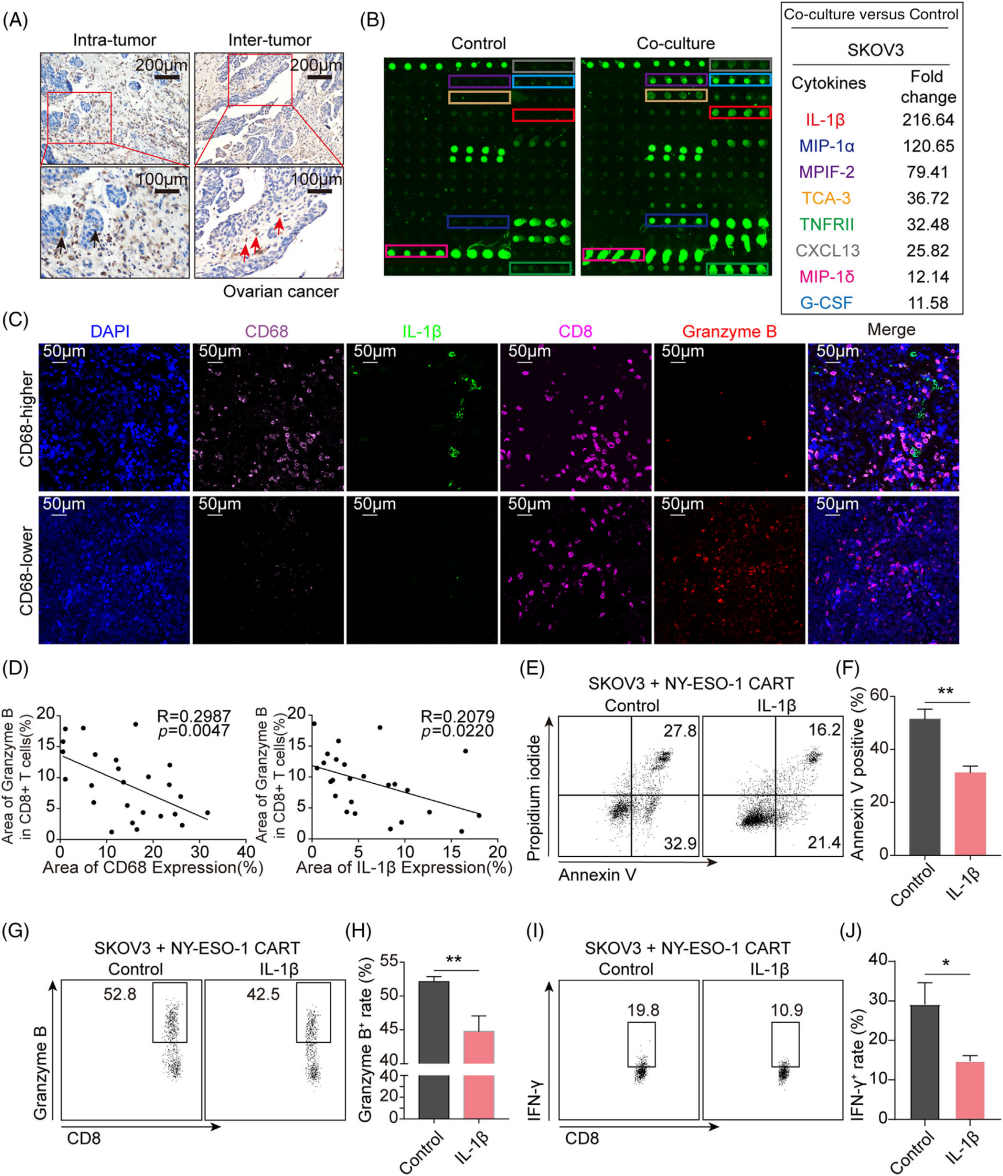
Identification of interleukin (IL)‐1β’s immunosuppressive phenotype. (A) Representative images of immunohistochemistry (IHC) staining of CD68^+^ macrophages (Mφ) in ovarian cancer sections showing the intra‐tumor Mφ (black arrows for intra‐tumor Mφ) and inter‐tumor Mφ (red arrows for inter‐tumor Mφ). (B) Representative images of immune‐related human inflammation array analysis of THP1‐derived Mφ (THP1 Mφ) and SKOV3 co‐cultured supernatant medium. (C) Representative images of fluorescence multiplex immunohistochemical analysis of CD68^+^ Mφ infiltration, IL‐1β secretion, CD8^+^ T cell infiltration, and granzyme B expression in tumor sections from patients with ovarian cancer. (D) Scatter plot of CD68^+^ Mφ infiltration/IL‐1β secretion and granzyme B expression levels of CD8^+^ T cells in tumors from patients with ovarian cancer (*n* = 5), five fields of each tissue were selected randomly. Data are assessed using linear regression analysis. (E–J) New York esophageal squamous cell carcinoma‐1 (NY‐ESO‐1) positive SKOV3 cells were incubated with or without IL‐1β for 72 h and then directly planted together with NY‐ESO‐1 chimeric antigen receptor T (CAR‐T) cells at a ratio of 1:2 for 5 h. Apoptosis of tumor cells was determined with annexin V and propidium iodide staining (E‐F), and CAR‐T cells’ functional assays were evaluated with granzyme B expression (G‐H) and interferon (IFN)‐γ expression (I‐J). Mean values ± SEM. *p < 0.05, **p < 0.01 by unpaired Student's *t*‐test.

To investigate whether tumor‐infiltrated Mφ could produce an immunosuppressive microenvironment in ovarian cancer, a representative “cold” tumor, we determined the CD68 expression, a classic marker of Mφ, in clinical tumor specimens of high‐grade serous ovarian cancer (HG‐SOC) patients with immunohistochemistry (IHC) staining (*n* = 41). The images revealed that Mφ were massively distributed at both inter‐tumor and intra‐tumor (Figure 1A) of ovarian cancer, and over 80% of ovarian cancer tissues held abundant Mφ distribution (*n* = 41; Figure S1A). Due to the high frequency of Mφ and the close spatial location between tumor cells and Mφ, we prospected that the crosstalk between the two might contribute to the immunosuppressive TME. We then used a human inflammation array to explore mediators of the communication by determining the components released when ovarian cancer cell line SKOV3 was co‐cultured with Phorbol‐12‐myristate‐13‐acetate (PMA)‐primed THP1 Mφ, a human leukemia monocytic cell line. IL‐1β was identified as one of the most abundant cytokines in the culture medium (Figure 1B). Similar findings were observed when other tumor cell lines, including the lung cancer cell line A549 and the glioma cell line U87MG, were co‐cultured with PMA‐primed THP1 Mφ (Figure S1B,C). To explore whether the increased IL‐1β contributed to immune suppression, a TCGA dataset was analyzed, and we found that IL‐1β was negatively correlated with T cell activation (Figure S1D). To further verify this notion, we examined CD68, IL‐1β, CD8, and granzyme B, a cytotoxic executor, in clinical tumor tissues of HG‐SOC patients with fluorescence multiplex immunohistochemical analysis. The results showed that both the frequency of Mφ and IL‐1β levels were negatively associated with granzyme B expression in CD8^+^ T cells (Figure 1C,D). Additionally, to verify whether IL‐1β could directly restrain T‐cells activation and function, the primary human T cells were activated with anti‐CD3/28 antibodies under the administration of different concentrations of IL‐1β. The result showed that no significant changes were directly triggered by IL‐1β on the expression of CXCR3, granzyme B, and IFN‐γ of T cells (Figure S1F–I). As anti‐CD3/CD28 antibodies activated T cells cannot specifically target tumor cells in vitro without tumor antigen presentation during their development and maturation, chimeric antigen receptor T (CAR‐T) cells were used as a model for their specific antitumor abilities. To evaluate whether IL‐1β could affect the tumor‐killing capacity of T cells via modulating tumor cells, SKOV3 cells that express neoantigen New York esophageal squamous cell carcinoma‐1 (NY‐ESO‐1) higher, compared to the other two ovarian cancer cell lines A2780 and OVCAR8 (Figure S1E), were pretreated with IL‐1β or without and then co‐cultured with NY‐ESO‐1 CAR‐T cells at a ratio of 1:2 to mimic the tumor‐specific killing of cytotoxic T cells. In comparison with non‐treatment SKOV3, IL‐1β‐primed SKOV3 tumor cells were more tolerant when directly co‐cultured with NY‐ESO‐1 CAR‐T cells (Figure 1E,F). The expression of granzyme B (Figure 1G,H) and IFN‐γ (Figure 1I,J) in CAR‐T cells was suppressed in the presence of IL‐1β. Taken together, these data illustrate that IL‐1β released when Mφ communicates with tumor cells induces immunosuppression without directly inhibiting functional cytokines expression of T cells.

### 2.2 IL‐1β secreted from Mφ enhances PD‐L1 expression in tumor cells

**FIGURE 2:**
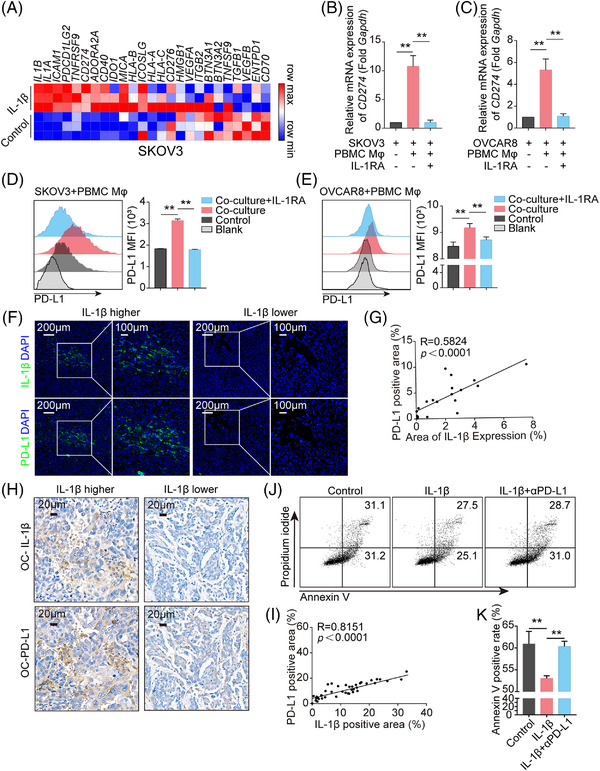
IL‐1β promotes tumor programmed death‐ligand 1 (PD‐L1) expression. (A) Heatmap showing relative mRNA expression of immune checkpoint signature genes in SKOV3 cell line. (B‐C) The *CD274* mRNA expression in SKOV3 and OVCAR8 cells after co‐culturing with peripheral blood mononuclear cell‐derived Mφ (PBMC Mφ) with or without IL‐1 receptor antagonist (IL‐1RA) pretreatment was determined using real‐time polymerase chain reaction (PCR; 48 h, *n* = 4). (D‐E). Representative histograms of MFI and bar charts showing membrane PD‐L1 expression in SKOV3 and OVCAR8 cells after 72 h incubation with PBMC Mφ with or without IL‐1RA pretreatment (*n* = 4). (F) Representative images of immunofluorescence (IF) staining of IL‐1β and PD‐L1 in ID8 ovarian cancer mouse tissues. (G) Scatter plot of IL‐1β secretion and PD‐L1 expression levels in ID8 ovarian cancer mouse tissues, three to five fields of each tissue were selected randomly. (H‐I) Representative images of IHC staining of IL‐1β and PD‐L1 in tumor tissues from patients with high‐grade serous ovarian cancer (HG‐SOC) (H); scatter plot of PD‐L1 and IL‐1β expression levels in tumor tissues from patients with HG‐SOC (*n* = 41), five fields of each tissue were selected randomly for calculating the mean value (I). (J‐K) NY‐ESO‐1‐positive SKOV3 cells were incubated with or without IL‐1β for 48 h and then directly planted together with NY‐ESO‐1 CAR‐T cells at a ratio of 1:2 for 5 h in the presence of anti‐PD‐L1 antibodies or not. Apoptosis of tumor cells was determined with annexin V and propidium iodide staining (J), and the ratio of annexin V‐positive tumor cells was quantified (K). All graphs show mean ± SEM. Data were assessed using an unpaired Student's *t*‐test or linear regression analysis. CSF, colony‐stimulating factor. OC, ovarian cancer. * *p* < 0.05; ** *p* < 0.01.

To further investigate the mechanisms underlying IL‐1β‐mediated immunosuppression, we focused on how IL‐1β reshaped the gene expression profile of tumor cells. With RNA‐sequencing, IL‐1β enhanced a range of immune inhibitory molecules expression on tumor cells, including *CD274*, *PDCD1LG2*, and *IDO1*. Among these molecules, the level of *CD274* gene, which encodes PD‐L1, was consistently elevated in all tumor cells, including the ovarian cancer cell line SKOV3 (Figure 2A), the lung cancer cell line A549 (Figure S1J), and the glioma cell line U87MG (Figure S1K). To explore whether the elevated PD‐L1 could be verified in vitro, primary human peripheral blood mononuclear cell‐derived Mφ (PBMC Mφ) were co‐cultured with human ovarian cancer cell lines, SKOV3 and OVCAR8, in the presence or absence of IL‐1 receptor antagonist (IL‐1RA). The results showed that PD‐L1 expression in tumor cells was upregulated, based on elevated mRNA and protein levels (Figure 2B–E). However, IL‐1RA blocked this upregulation (Figure 2B–E). Similar results were observed in PMA‐primed THP1 Mφ co‐culturing with SKOV3/OVCAR8/A549/U251/U87MG (Figure S2A–O). To further validate the IL‐1β‐PD‐L1 axis, mouse tumor tissues were employed to stain IL‐1β and PD‐L1. The results showed a positive correlation between PD‐L1 expression and IL‐1β secretion in mouse ovarian cancer ID8 tumor tissues (Figure 2F,G) and mouse lewis l LLC tumor tissues (Figure S3A,B). Consistent with this, IHC staining with anti‐IL‐1β and anti‐PD‐L1 in 41 surgically resected human ovarian cancer samples demonstrated that IL‐1β was abundant in tumor tissues and positively correlated with the level of PD‐L1 (Figure 2H,I). In addition, IL‐1β treatment triggered dose‐dependent increases in PD‐L1 expression in SKOV3, A549, U251, and U87MG cells directly (Figure S4A–D). Importantly, a neutralizing antibody targeting PD‐L1 dramatically normalized the cytotoxicity of NY‐ESO‐1 CAR‐T cells suppressed in the presence of IL‐1β (Figure 2J,K). Thus, these results confirmed that IL‐1β induced an immunosuppressive feature via improving PD‐L1 expression.

To determine whether IL‐1β was secreted by Mφ or tumor cells, we knocked down IL‐1β in tumor cells in the co‐culture system (Figure S5A–D). With real‐time polymerase chain reaction (PCR) and flow cytometry detection, *CD274* mRNA and its protein levels before and after IL‐1β knockdown were similar in all four cell lines (Figure S5E–P), which revealed that the IL‐1β released in the co‐culture medium was dominantly derived from Mφ. To validate the notion in tumor tissues, the images of the above fluorescence multiplex immunohistochemical analysis were reused to analyze IL‐1β distribution. The results showed that more than 80% of IL‐1β was co‐localized with CD68‐positive Mφ (Figure S6A,B). Consistently, the majority of IL‐1β was expressed in F4/80‐positive Mφ in ID8 and LLC mouse tumor tissues with immunofluorescence (IF) staining (Figure S6C–E).

Taken together, these results demonstrate that IL‐1β is secreted by Mφ and is pivotal in regulating PD‐L1 expression by tumor cells in the TME.

### 2.3 Nuclear factor‐κB (NF‐κB) is involved in the upstream signaling of PD‐L1 expression following IL‐1β stimulation

**FIGURE 3:**
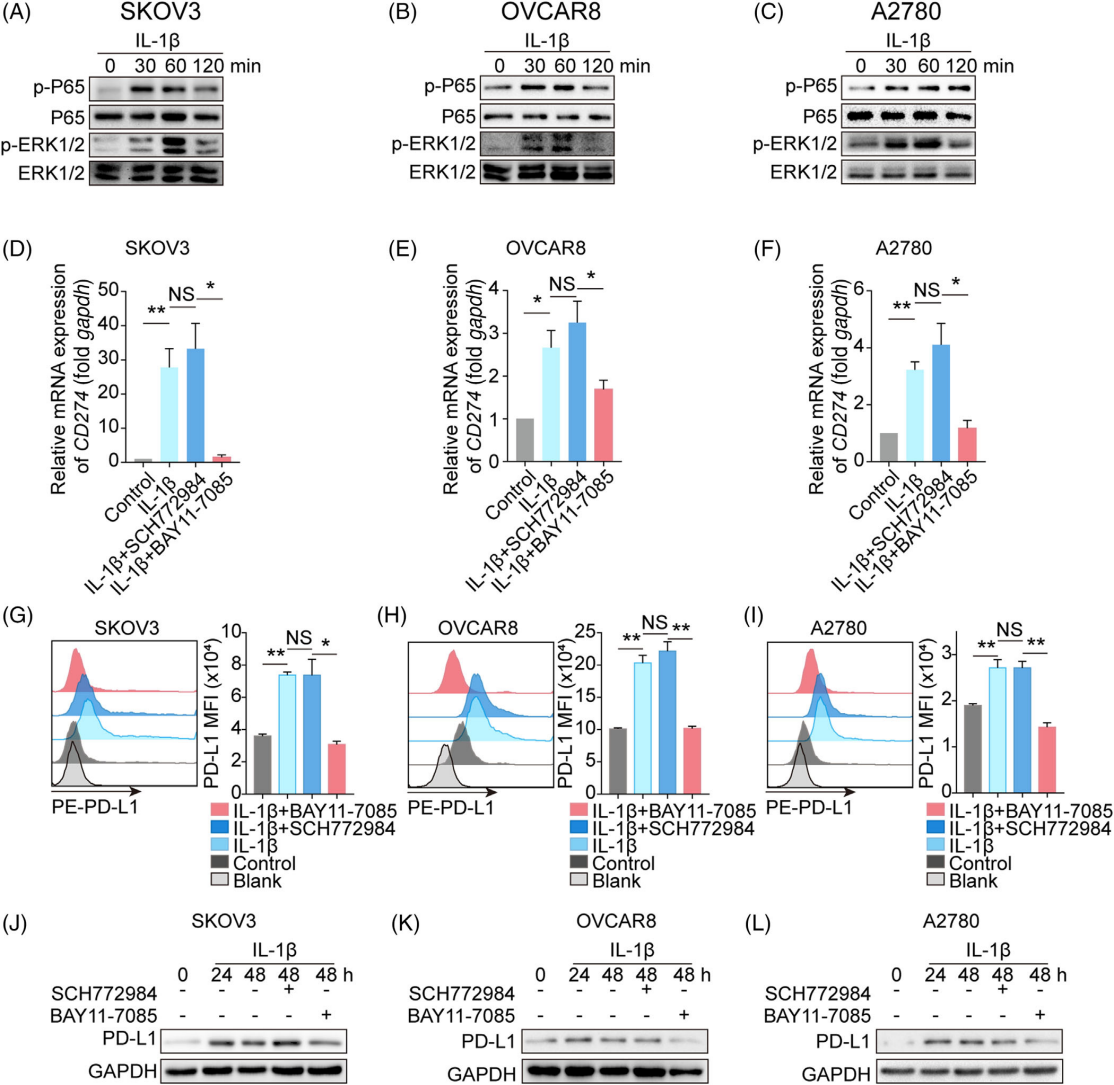
IL‐1β activates nuclear factor‐κB (NF‐κB) signaling to increase PD‐L1 expression in tumor cells. (A–C) Ovarian cancer cell lines, including SKOV3, OVCAR8, and A2780, were treated with IL‐1β (10 ng/mL) for indicated durations, and the protein levels of P65, p‐P65, ERK1/2, and p‐ERK1/2 were measured using western blotting. (D–F) Tumor cells were treated with IL‐1β (10 ng/mL) for 8 h in the presence or absence of pretreatment with BAY11‐7085 (NF‐κB inhibitor, 5 μM) and SCH772984 (mitogen‐activated protein kinase (MAPK) signaling inhibitor, 10 μM), *n* = 4. The *CD274* mRNA expression was measured using real‐time PCR (*n* = 4). G‐I. Representative histograms of mean fluorescence intensity (MFI) and bar charts showing membrane PD‐L1 expression after IL‐1β treatment with or without pretreatment with BAY11‐7085 (NF‐κB inhibitor, 5 μM) and SCH772984 (MAPK signaling inhibitor, 10 μM), n = 4. J‐L. Tumor cells were treated with IL‐1β (10 ng/mL) for indicated durations in the presence or absence of pretreatment with BAY11‐7085 (NF‐κB inhibitor, 5 μM) and SCH772984 (MAPK signaling inhibitor, 10 μM). The PD‐L1 expression was measured using western blotting. All graphs show mean ± SEM. Data were assessed using an unpaired Student's *t*‐test. * *p* < 0.05; ** *p* < 0.01. NS, no significance.

Next, we sought to explore the mechanism underlying the IL‐1β‐induced increase in PD‐L1 expression. NF‐κB and mitogen‐activated protein kinase/extracellular regulated protein kinase (MAPK/ERK) involved in the regulation of PD‐L1 expression have been identified as two major signaling pathways downstream of IL‐1β.^24^, ^25^, ^26^ Thus, we asked whether NF‐κB or MAPK/ERK signaling pathway contributed to the IL‐1β‐PD‐L1 axis. Three ovarian cancer cell lines SKOV3, OVCAR8, and A2780 were administrated with or without IL‐1β, and we found that IL‐1β increased the phosphorylation of P65 (NF‐κB activation) and ERK, beginning at 30 min after treatment, compared to non‐treatment groups (Figure 3A–C). Furthermore, pharmacologic inhibition of NF‐κB activation (BAY11‐7085) dampened the IL‐1β‐induced upregulation of PD‐L1 transcription in all three tumor cell lines, while ERK inhibition (SCH772984) did not work (Figure 3D–F). Consistent with the transcription, the cell membrane (Figure 3G–I) and total protein (Figure 3J–L) of PD‐L1 were reduced following treatment with the NF‐κB inhibitor but not with the ERK inhibitor. Finally, A549, U251, and U87MG were used to verify the essential role of NF‐κB in the IL‐1β‐induced PD‐L1 elevation (Figure S7A–L). Collectively, we propose that NF‐κB functions as a key mediator in the IL‐1β‐induced PD‐L1 expression.

### 2.4 Tumor cell‐secreted lactate is critical for the expression of IL‐1β by Mφ

**FIGURE 4:**
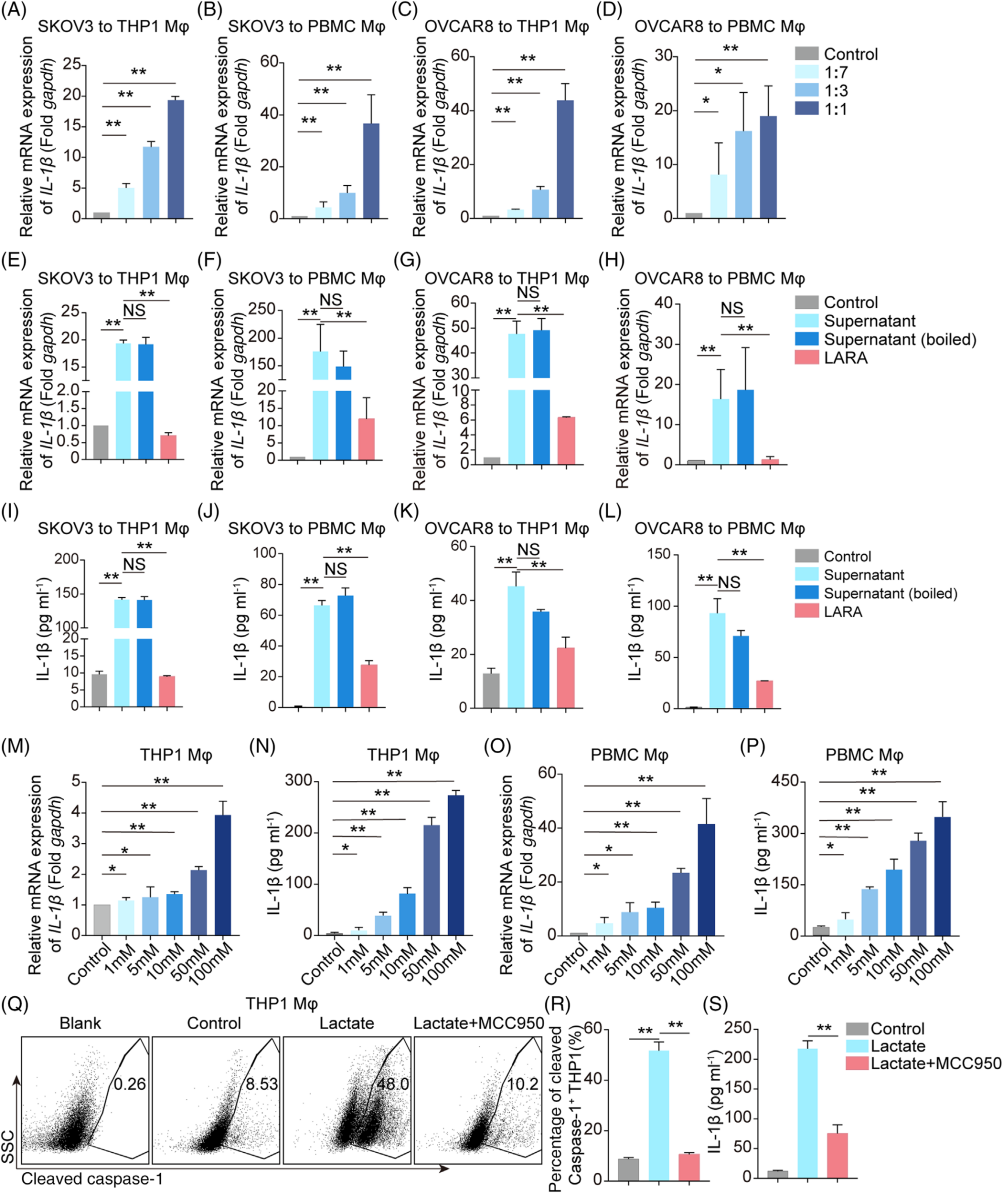
Lactate increases the transcription and secretion of IL‐1β in Mφ. (A–D) SKOV3 and OVCAR8 supernatant mixed with fresh medium at ratios of 1:1, 1:3, or 1:7 were used to treat THP1 Mφ and PBMC Mφ for 8 h, and then the *IL‐1β* mRNA expression in Mφ was determined using real‐time PCR (*n* = 4). (E–L) THP1 Mφ and PBMC Mφ were treated with SKOV3 or OVCAR8 supernatant or boiled supernatant (100°C for 5 min) mixed with fresh medium at a 1:1 ratio in the presence (red) or absence of lactate receptor antagonist (LARA) for about 8 h to determine the *IL‐1β* mRNA expression in Mφ using real‐time PCR (E–H) and for about 72 h to determine the IL‐1β concentration in the supernatant using enzyme linked immunosorbent assay (ELISA) (I–L). (M–P) THP1 Mφ and PBMC Mφ were treated with different proportions of sodium lactate for about 6 h to measure the *IL‐1β* mRNA expression in Mφ using real‐time PCR (M‐N) and for about 96 h to determine IL‐1β secretion in the supernatant using ELISA (O‐P). (Q‐R) Representative flow plots and a bar chart showing cleaved caspase‐1 in THP1 Mφ treated with lactate with or without MCC950 (*n* = 4). (S) IL‐1β secretion in the supernatant of THP1 Mφ treated with lactate with or without MCC950 (*n* = 4). All graphs show mean ± SEM. Data were assessed using an unpaired Student's *t*‐test. * *p* < 0.05; ** *p* < 0.01. NS, no significance.

Although IL‐1β secreted by Mφ upregulates PD‐L1 expression in tumor cells, the molecule that induces IL‐1β secretion from Mφ is still unknown. Thus, we sought to determine whether tumor cells can trigger IL‐1β secretion from Mφ. SKOV3 and OVCAR8 tumor culture medium (TCM) and fresh medium were mixed in different ratios and used to culture THP1/PBMC Mφ. The results showed that the transcription of IL‐1β was increased by TCM in a dose‐dependent manner (Figure 4A–D), hinting that there were functional molecules, including proteins, nucleic acids, or other metabolites, that acted as inducers. More interestingly, we showed that boiled TCM (inactivated protein and nucleic acid) still facilitated IL‐1β transcription and secretion (Figure 4E–L), suggesting that the molecules responsible for inducing IL‐1β expression were secreted metabolites rather than proteins or nucleic acids. Surprisingly, pretreatment with a lactate receptor antagonist (LARA) suppressed IL‐1β transcription and secretion (Figure 4E–L). Analysis of the TCM from A549, U251, and U87MG cells corroborated these findings (Figure S8A–I). Other metabolite receptor antagonists, including AhR, mGluR5, and A2AR inhibitors had little effect on IL‐1β expression (Figure S9A,B). These results revealed that lactate was a potential candidate associated with boosting IL‐1β expression. Of note, we found that IL‐1β transcription and secretion were increased in a dose‐dependent manner by direct lactate treatment (Figure 4M–P). Further investigation revealed that pro‐caspase‐1 was cleaved under lactate stimulation, while treatment with an NOD‐like receptor thermal protein domain accociated protein 3 (NLRP3) inflammasome inhibitor MCC950 weakened this cleavage and decreased the secretion of IL‐1β from THP1 Mφ (Figure 4Q–S). Taken together, we elucidate that tumor cell‐derived lactate enhances IL‐1β transcription and secretion by Mφ.

### 2.5 IL‐1β‐induced C‐C motif chemokine ligand 2 (CCL2) recruits Mφ

**FIGURE 5:**
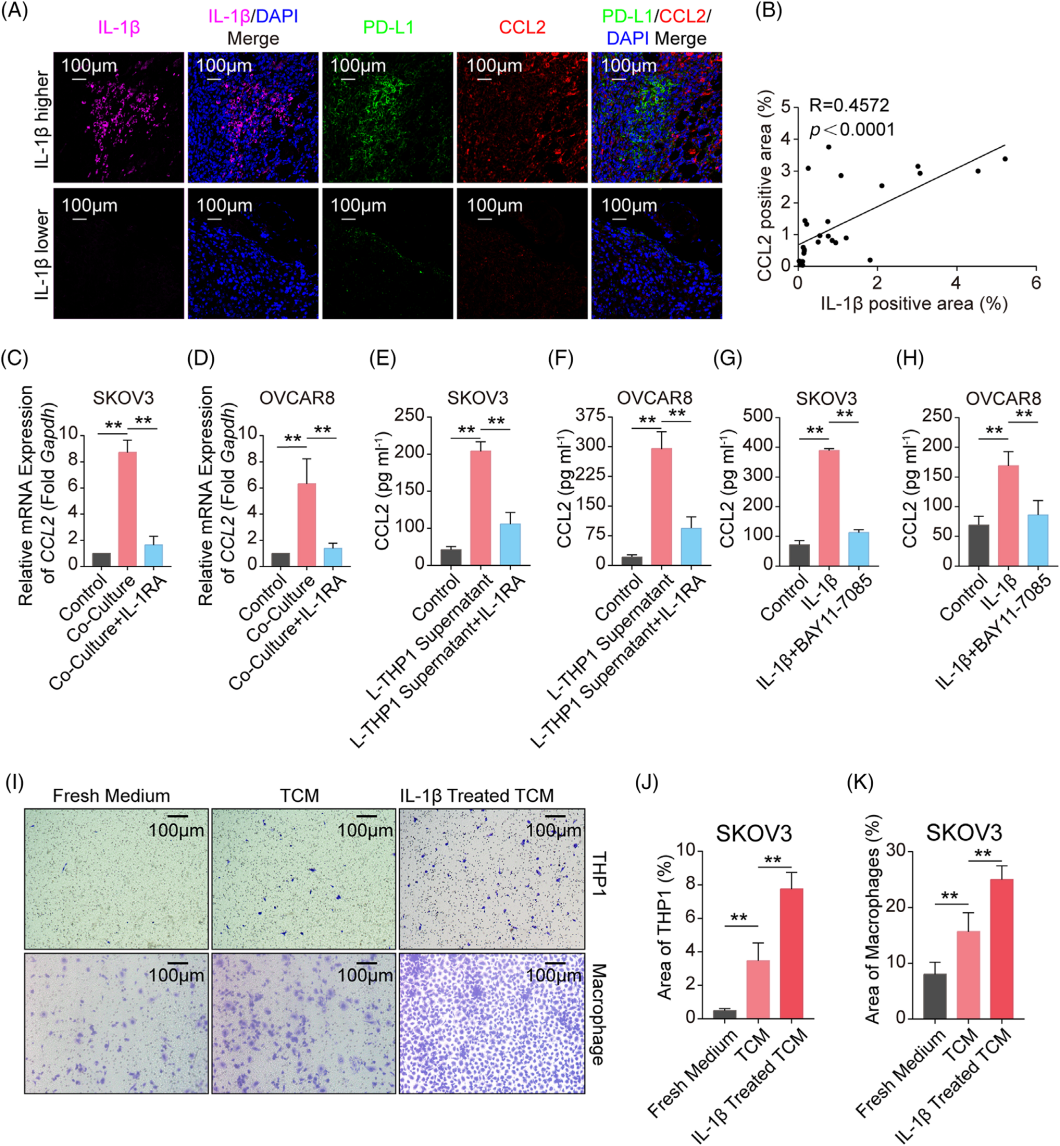
IL‐1β secreted by Mφ promotes tumor C‐C motif chemokine ligand 2 (CCL2) expression and Mφ infiltration. (A) Representative images of IF staining with serial sections of ovarian cancer mouse tissues. IL‐1β (purple) and DAPI were co‐stained in one section (left two); PD‐L1, CCL2, and DAPI were co‐stained in another section (right three). (B) Scatter plot of IL‐1β and CCL2 secretion levels in ovarian cancer mouse tissues, five fields of each tissue were selected randomly. (C‐D) SKOV3 and OVCAR8 cell lines were co‐cultured with THP1 Mφ in the presence or absence of pretreatment with IL1RA, and then the *CCL2* mRNA expression in tumor cells was measured using real‐time PCR. E‐F. The CCL2 concentration in SKOV3 and OVCAR8 culture medium was measured using ELISA after the two cells were treated with THP1 Mφ supernatant activated with lactate (L‐THP1 supernatant) in the presence or absence of IL1RA. G‐H. The CCL2 concentration in the SKOV3 and OVCAR8 culture medium was measured using ELISA after the two cells were treated with IL‐1β in the presence or absence of pretreatment with BAY11‐7085 (NF‐κB inhibitor, 5 μM). (I) Representative images of migration assays of THP1 Mφ and PBMC Mφ in fresh medium or tumor culture medium (TCM) or IL‐1β‐activated TCM from SKOV3. (J‐K) Bar charts of migration assays of THP1 Mφ (J) and PBMC Mφ (K) in fresh medium or TCM or IL‐1β‐activated TCM from SKOV3. Data were assessed by unpaired Student's *t*‐test or linear regression analysis. ** *p* < 0.01.

We figured out a loop composed of tumor cells, lactate, Mφ, IL‐1β, and PD‐L1 above. What is more, the loop could be deteriorated by the replenishment of Mφ in the TME. Therefore, we further assessed the role of secreted IL‐1β in promoting Mφ infiltration in TME. As CCL2 functions as the most critical chemokine in the recruitment of Mφ to tumor sites,^27^, ^28^, ^29^ we analyzed transcriptome data from IL‐1β‐treated A549/ U87MG cells and found that *CCL2* was one of the most highly upregulated chemokine genes (Figure S10A,B). This was confirmed by the observation of a significant positive correlation between IL‐1β and CCL2 levels in ID8 tumor tissues (Figure 5A,B) and LLC tumor tissues (Figure S12A,B). To further clarify whether Mφ‐derived IL‐1β induced CCL2 expression, SKOV3/OVCAR8 and THP1 Mφ were co‐cultured in the presence or absence of IL‐1RA. The results showed that co‐culturing with THP1 Mφ strongly increased CCL2 expression in tumor cells, and this elevation could be restricted under the treatment of IL‐1RA (Figure 5C,D). Furthermore, a lactate‐primed THP1 Mφ culture medium was used to stimulate SKOV3/OVCAR8 in the presence or absence of IL‐1RA. We found that CCL2 secretion declined following the administration of IL‐1RA, compared to the treatment with THP1 Mφ supernatant alone (Figure 5E,F). Furthermore, inhibition of NF‐κB by BAY11‐7085 blocked the IL‐1β‐induced CCL2 secretion from tumor cells in different types of cancer cells (Figures 5G,H andS11A–C). Last, transwell migration assays showed that IL‐1β‐primed SKOV3‐TCM significantly increased the recruitment of THP1/PBMC Mφ, compared to control TCM and fresh medium (Figure 5I–K), and similar results were obtained using IL‐1β‐primed U87MG‐TCM (Figure S12C–E). Taken together, these results show that IL‐1β secreted by Mφ stimulates CCL2 secretion in tumor cells, which in turn boosts Mφ infiltration, thus further increasing PD‐L1 expression in tumor cells and orchestrating tumor immunosuppressive microenvironment.

### 2.6 Blockade of IL‐1β inhibits tumor growth and reverses the tumor immunosuppression

**FIGURE 6:**
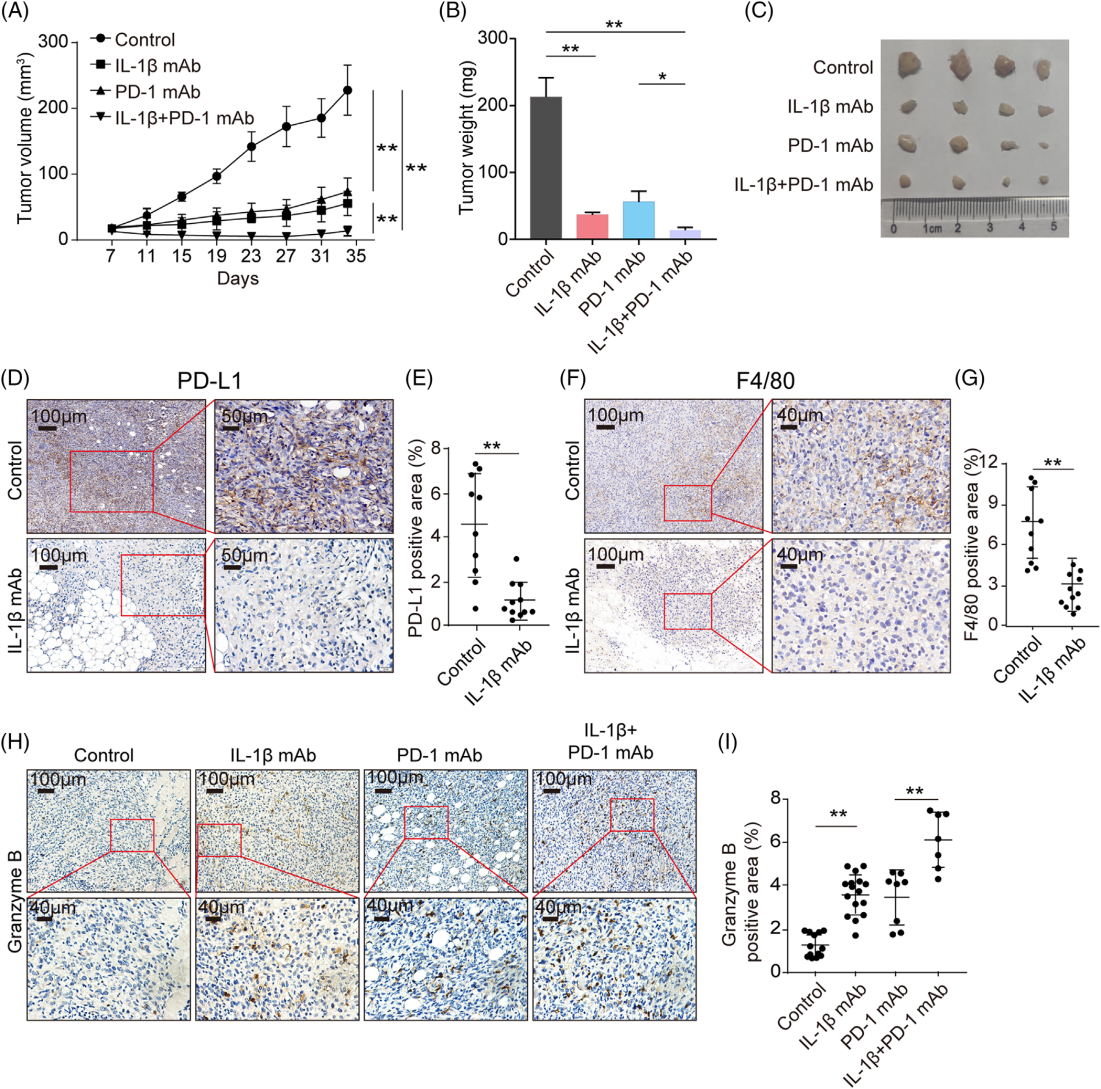
Anti‐IL‐1β mAb exhibits antitumor effects mono‐therapeutically or in combination with anti‐programmed death‐1 (PD‐1) mAb in an ID8 mouse model. (A) Tumor volume of control mice (●), mice treated with anti‐IL‐1β monoclonal antibody (mAb) (■), mice treated with anti‐PD‐1 mAb (▲), and mice treated with both anti‐IL‐1β mAb and anti‐PD‐1 mAb (▼) in the ID8 ovarian cancer mouse model. (B) Weights of ID8 tumor tissues. (C) A photo of ID8 tumor tissues. (D) Representative images of IHC staining of PD‐L1 in the ID8 mouse model. (E) Percentage of PD‐L1 positive area in tumor sections from control and anti‐IL‐1β mAb groups, and two to four fields of each tumor section were selected randomly for calculating the ratios of positive area. (F) Representative images of IHC staining of F4/80^+^ Mφ in tumor sections from the ovarian cancer mouse model, and two to four fields of each tumor section were selected randomly for calculating the ratios of positive area. (G) Percentage of F4/80^+^ Mφ positive area in tumor sections from control and anti‐IL‐1β mAb groups. (H) Representative images of IHC staining of granzyme B in mouse cancer sections from the ID8 mouse model. (I) Percentage of the granzyme B positive area in tumor sections from the indicated treatment groups, and three to five fields of each tumor section were selected randomly for calculating the ratios of positive area. All graphs show mean ± SEM. Data were assessed with unpaired Student's *t*‐test. * *p* < 0.05; ** *p* < 0.01.

The above data clarified mechanisms underlying the IL‐1β‐centered immunosuppressive loop. Next, we asked whether the IL‐1β blockade could reinvigorate the suppressed immune responses and synergistically inhibit the tumor growth with ICB in syngeneic mice models. To this end, immunocompetent C57BL/6 mice were subcutaneously injected with mouse ovarian cancer cell line ID8 under general anesthesia and administrated with anti‐IL‐1β neutralizing antibodies or isotype immunoglobulin G (IgG) twice a week. The mice were then injected with or without anti‐PD‐1 antibodies from Day 7. As expected, tumor growth was sloped down following anti‐IL‐1β antibodies treatment, similar to the administration with anti‐PD‐1 antibodies (Figure 6A–C). Interestingly, a combination of IL‐1β and PD‐1 blockade showed a better antitumor effect than monotherapy (Figure 6A–C). Subsequently, to assess how IL‐1β blockade reshaped the TIME, we determined the levels of PD‐L1, F4/80, and granzyme B in ID8 tumor tissues with IHC staining. The results showed that IL‐1β blockade significantly decreased the expression of PD‐L1 in tumor tissues (Figure 6D,E), and the frequency of Mφ was much less in anti‐IL‐1β antibody‐treated tumor tissues, compared to non‐treatment (Figure 6F,G). Moreover, both the blockade of IL‐1β and PD‐1 promoted the expression of granzyme B in mice tissues, and the combinational treatment achieved higher levels of granzyme B than monotherapy (Figure 6H,I). In addition, a synergistic antitumor effect was achieved when combining the IL‐1β and PD‐1 blockade in the LLC tumor‐bearing mouse model (Figure S13A–C), and anti‐IL‐1β antibodies also reversed the suppressive TIME of LLC tumors (Figure S13D–I). Collectively, IL‐1β is a promising therapeutic target when used in combination with ICB molecules due to its role in the malignant loop.

### 2.7 IL‐1β is positively associated with the expression of PD‐L1 and CCL2, while negatively associated with disease progression in human public databases

We next verified the modulatory effect of IL‐1β on PD‐L1 expression and the formation of the malignancy loop with bioinformatic analysis. Three Gene Expression Omnibus datasets were analyzed and revealed that IL‐1β levels were positively correlated with PD‐L1 and CCL2 expression in ovarian cancer, NSCLC, and glioma (Figure S14A,B,D,E,G,H). Higher levels of IL‐1β were correlated with poorer prognosis in patients with ovarian cancer after 5 years of follow‐up (Figure S14C). IL‐1β levels in tumor tissues were negatively correlated with overall survival in patients with other two types of cancers, which was observed with Gene Expression Omnibus datasets (Figure S14F,I). Additionally, TCGA data about liver hepatocellular carcinoma, pancreatic ductal adenocarcinoma, and stomach adenocarcinoma were analyzed to confirm these results (Figure S15A–I). Moreover, a signature of negative regulation of IL‐1β production was enriched in the ICB‐responded lung cancer patients (Figure S16A) in Gene Expression Omnibus datasets (GSE126044). The relationship between IL‐1β and PD‐L1/CCL2 was further verified in patient‐derived datasets, uncovering that enhancing therapeutic efficacy and sensitivity by combining anti‐IL‐1β with ICB was necessary for improving the current poor responsiveness of anti‐PD‐1/L1 treatment in solid tumors.

## 3 DISCUSSION

**FIGURE 7:**
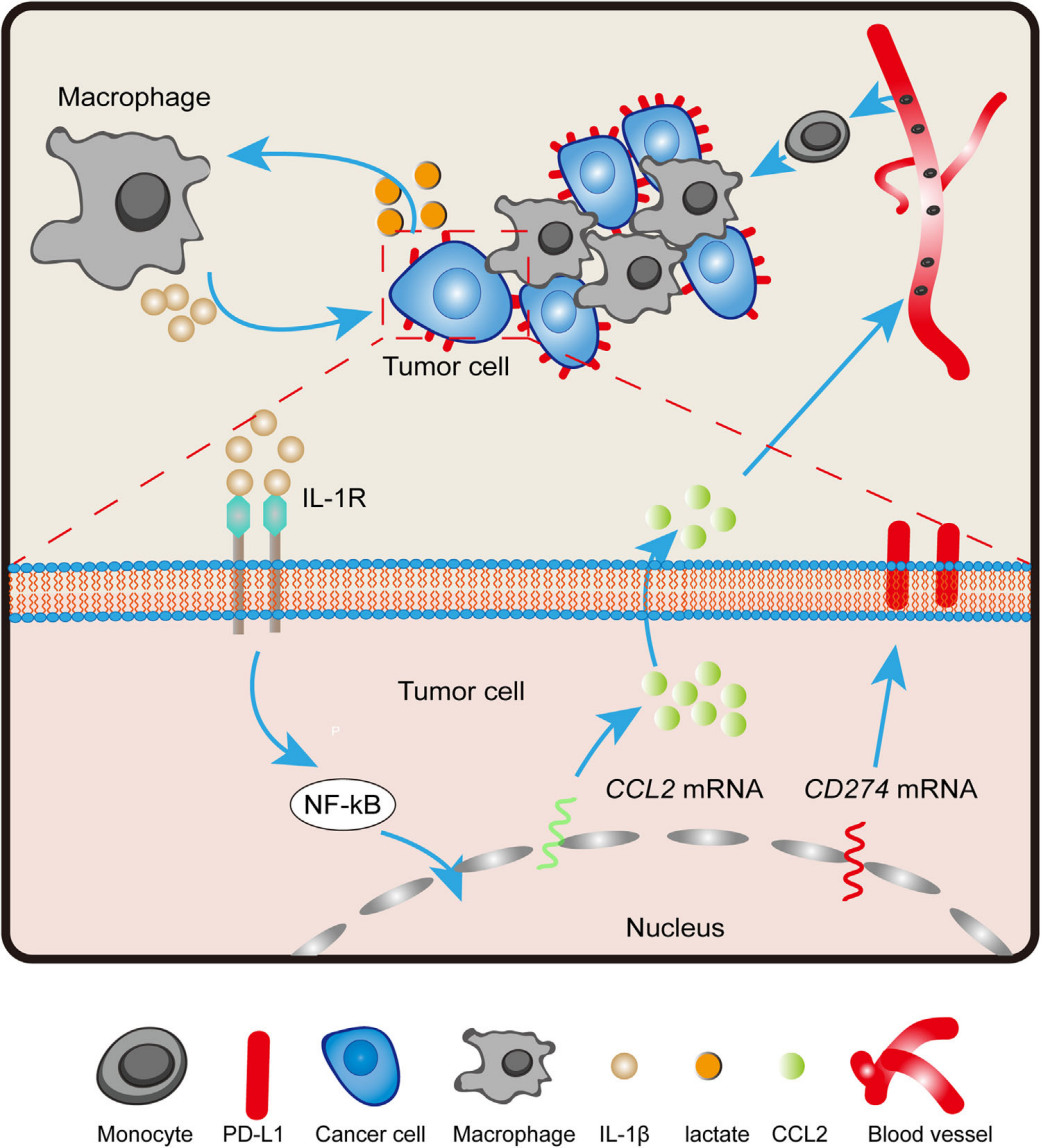
Crosstalk between tumor cells and Mφ results in immunosuppressive TME and Mφ accumulation.

TAMs are widely regarded as immunosuppressive members, functioning inter‐tumor and intra‐tumor by expressing abundant immune checkpoint ligands and secreting cytokines, which then directly or indirectly weaken antitumor immune activity.^7^, ^8^, ^9^, ^11^, ^12^, ^13^, ^30^, ^31^ Here, we found that the pro‐inflammatory cytokine IL‐1β could be triggered and released from Mφ by lactate derived from tumor cells. IL‐1β, in turn, drove an immune inhibitory phenotype on tumor cells, especially on the increase in PD‐L1, by activating the NF‐κB signaling pathway. Thus, tumor‐released lactate and Mφ‐derived IL‐1β mediated the crosstalk loop and caused immunosuppression. Moreover, activated NF‐κB signaling promoted CCL2 secretion in tumor cells, leading to further monocyte recruitment and replenishment of the Mφ population, which maintained the malignant feedback loop (Figure 7). The in vivo administration of the anti‐IL‐1β antibody enhanced the therapeutic efficacy of anti‐PD‐1 in tumor cell‐derived xenograft models.

Modulation of the immune microenvironment by Mφ has been the focus of cancer research and clinical therapies, particularly those involving combining TAM with ICB in the treatment of multiple solid tumors. For example, blocking CSF1 or CSF1R to impair TAMs has been shown to enhance the therapeutic efficacy of anti‐PD‐1 or anti‐CTLA4 antibodies since the CSF1‐CSF1R axis contributes to the maturation and differentiation of Mφ.^32^, ^33^, ^34^ Alternatively, the replenishment of Mφ derived from circulating monocytes is also essential to the function of TAMs. Therefore, CCR2 and CCR5 antagonists can dramatically improve the antitumor effect of immunotherapies.^35^ Thus, TAM‐targeted therapies combined with ICB are a promising research area, and their detailed mechanisms have been well studied. However, the crosstalk between Mφ and tumor cells to modulate the TIME needs further investigation. Here, we co‐cultured THP1 Mφ with different kinds of tumor cells to identify the molecules released into the supernatant. Interestingly, IL‐1β was one of the most abundant molecules in the co‐culture supernatant. Importantly, upregulated IL‐1β expression was associated with attenuated cytotoxicity of CAR‐T cells and reduced CD8^+^granzyme B distribution in patient‐derived tumor tissues. The immunosuppressive role of IL‐1β was due to its upregulation of immune‐suppressing genes in different tumor cells, with *CD274* being the most consistently upregulated gene. Additionally, IL‐1β has been reported to upregulate PD‐L1 expression in hepatocellular carcinoma cells,^36^ while how the IL‐1β and the elevated PD‐L1 modulated the TIME remains elusive. Here, we proposed that IL‐1β involved in the interplay between tumor cells and TAMs elicited immunosuppression.

The Food and Drug Administration (FDA) has approved anti‐PD‐1 and anti‐PD‐L1 antibodies for the treatment of tumors with high tumor mutation burden, microsatellite instability, abundant cytotoxic T lymphocyte infiltration, or increased PD‐L1 expression levels.^37^ PD‐L1 expression levels in tumors affect the outcome of anti‐PD‐1/L1 therapy. PD‐L1 is ubiquitously expressed and significantly upregulated under the education of multiple TME components.^37^, ^38^ IFN in the TME has been identified as the most critical agonist responsible for inducing PD‐L1 expression. However, IFN acts as a double‐edged sword and can also dramatically fuel both innate and adaptive immune responses to tumor aggressiveness.^39^ Therefore, patients with malignant tumors may gain little benefit from direct treatment with IFN, while the lack of IFN in the TME can also result in poor ICB efficacy.^39^ Thus, IFN is an un‐effective therapeutic target for enhancing antitumor immune responses.

Recent studies focusing on breast cancer and renal cell carcinoma have shown that neutralizing IL‐1β can rescue the immunosuppressive microenvironment and work synergistically to block PD‐1, but the detailed mechanisms need to be further explored.^40^, ^41^ In this study, we found that IL‐1β significantly promoted the expression of PD‐L1 in multiple cancers, including ovarian cancer, lung cancer, and glioma. The detailed mechanisms underlying PD‐L1 expression in tumor cells were well‐studied such as STAT3, MYC, HIF, BRD4, NF‐κB, c‐Jun, TFEB, and so on.^38^, ^42^ Here, owing to the NF‐κB signaling pathway being a classic downstream of the IL‐1β‐ IL‐1β receptor axis, we identified that the activation of NF‐κB triggered by IL‐1β contributed to the increase of PD‐L1 expression. Meanwhile, as a proinflammatory cytokine, IL‐1β could elicit a wide range of chemokines expression to orchestrate the TIME. In oral cancer, IL‐1β can trigger tumor cells to secret CXCL1,^43^ and CXCL1 is closely associated with the infiltration of MDSCs.^44^ IL‐1β can also facilitate CCL20 expression,^45^ and the CCL20–CCR6 axis participates in the recruitment of Tregs and T helper 17 cells.^46^ Here, we found that CCL2 was the most highly enhanced chemokine induced by IL‐1β with RNAseq data and facilitated infiltration of Mφ. The blockade of IL‐1β not only impaired PD‐L1 expression in tumor cells but also decreased in vivo Mφ infiltration. An anti‐IL‐1β antibody has been approved by the FDA for the treatment of autoimmune disorders such as systemic juvenile idiopathic arthritis and adult‐onset Still's disease, and combining anti‐IL‐1β and anti‐PD‐1/L1 antibodies potentiates treatment effects in clinical applications.

Generally, tumor‐infiltrating Mφ tends to be polarized to M2 Mφ that presents as a pro‐tumoral phenotype and releases VEGF, TGF‐β, and IL‐10, which promote angiogenesis and immunosuppression, as well as facilitate tumor progression.^47^ Using co‐culture systems with IL‐1β receptor inhibitors or tumor cells with the *Il‐1β* gene knocked down, we discovered that the upregulated IL‐1β was derived from Mφ. Thus, we demonstrated that IL‐1β, a well‐known marker of M1 Mφ, could also be released by TAMs, which were usually viewed as M2 Mφ educated by mounts of elements in the TME.^48^ Moreover, IL‐1β, a pro‐inflammatory cytokine, is secreted upon activation of inflammasomes such as NLRP1, NLRP3, NLRC4, and AIM2.^49^ However, the detailed mechanisms underlying inflammasome activation in TAMs, triggered by molecules associated with tumor cells, are unclear. The Warburg effect is the most characteristic metabolic reprogramming of tumor cells, with lactate being the primary metabolite released from tumor cells.^50^ However, the modulation of lactate in the activation of the inflammasome is controversial.^51^, ^52^ For example, lactate can suppress lipopolysaccharide (LPS)‐induced IL‐1β secretion.^53^ However, lactate also contributes to the Nigericin‐induced activation of inflammasome.^54^ Here, we found that lactate, the aerobic glycolysis byproduct released by tumor cells, can directly induce NLRP3 inflammasome activation and promote IL‐1β maturation. Additionally, lactate is the initial driving factor that triggers the malignant loop for deteriorating the TME.

It is well‐established that IFN is primarily secreted by activated T cells in “hot” tumors and strongly enhances the PD‐L1 expression,^55^ while ovarian cancer is a classic type of “cold” tumor,^56^ which might infer a poor PD‐L1 expression. However, about half of the ovarian cancer patients exhibit a high expression of PD‐L1,^57^, ^58^ hinting other mechanisms to upregulate PD‐L1 expression should exist and need to be further explored. Our data illustrated that IL‐1β released from Mφ facilitated the PD‐L1 expression, which might uncover a critical mechanism in the elevation of PD‐L1 in “cold” tumors such as ovarian cancer. Moreover, the malignant loop between Mφ and tumor cells elicited a constant expression of IL‐1β and upregulation of PD‐L1, which might contribute to T‐cell exhaustion and desensitization of ICB.^59^, ^60^ Meanwhile, IL‐1β induced CCL2 secretion from tumor cells and exhibited a strong ability to recruit Mφ into the tumor site. The TAMs usually function as immune inhibitory cells to shape immunosuppressive TME and are closely associated with ICB resistance.^61^ Taken together, IL‐1β plays a central role in the attenuation of ICB via orchestrating immune hyporesponsiveness, which is similar to the notion that sustained IFN signaling induces tumor resistance to ICB,^60^ suggesting a strong rationale to combine ICB with IL‐1β blockade.

Collectively, we show that tumor‐released lactate could trigger IL‐1β secretion from Mφ by activating the NLRP3 inflammasome. Meanwhile, Mφ‐derived IL‐1β restrains immune responses by modulating both the PD‐L1 levels in tumor cells and the abundance of Mφ within TME, leading to the deterioration of the TIME. This feedback effect results in the immunosuppressive malignant loop. IL‐1β blockade suspends this loop and improves the therapeutic efficacy of anti‐PD‐1 antibodies.

## 4 MATERIALS AND METHODS

### 4.1 Cells

The ID8 cell line was a kind gift from the University of Kansas Medical Center. LLC, THP1, and SKOV3 cell lines were purchased from the National Collection of Authenticated Cell Cultures (Shanghai, China). The A2780, A549, U251, and U87MG cell lines were purchased from the American Type Culture Collection (Rockville). The OVCAR8 cell line was purchased from American Type Culture Collection. All cell lines were cultured following the manufacturer's guidelines.

### 4.2 Cell culturing

A549 (human lung cancer cell line), U251 (human glioma cell line), U87MG (human glioma cell line), LLC (mouse lung cancer cell line), and ID8 (mouse ovarian cancer cell line) cells were cultured in Dulbecco's modified Eagle's medium (DMEM; Gibco, 11965092). SKOV3 (human ovarian cancer cell line) cells were cultured in McCoy's 5A (modified) medium (Gibco, 12330031). THP1 (human monocyte cell line), A2780 (human ovarian cancer cell line), and OVCAR8 (human ovarian cancer cell line) cells were cultured in Roswell Park Memorial Institute (RPMI) 1640 medium (Gibco, 11875176). All media were supplemented with 10% fetal bovine serum (FBS) (Gibco, 10099141), and 100 μg/mL penicillin‐streptomycin (Gibco, 15070063). All cells were maintained at 37°C in a humidified chamber with 5% CO_2_.

### 4.3 Mice

Wild‐type C57BL/6 female mice (6 weeks old, healthy) were obtained from Beijing HFK Biotechnology Co. Ltd. The mice were housed under pathogen‐free conditions in the animal facilities of the Tongji Hospital of Huazhong University of Science and Technology (HUST). The study was approved by the ethics committee of animal experiments of Tongji Medical College. Eight‐week‐old female mice were used in all experiments.

### 4.4 Generation of primary human Mφ

Primary peripheral blood monocytes were isolated from the peripheral blood of healthy donors recruited from the Cancer Biology Research Center of Tongji Hospital using Ficoll density gradient centrifugation. Briefly, peripheral venous blood was added to the upper layer of Ficoll solution in a ratio of 1:1 and centrifuged at 800 g × 20 min, after that, the PBMCs layer in the mixture was washed with phosphate buffer saline (PBS) three times at 500 g × 10 min. RBCs were depleted with ACK Lysis Buffer at 4°C for 5 min. PBMCs were collected and washed with PBS three times before being plated into a culture dish. Monocytes were cultured for 6 days in DMEM (Gibco, 11965092) supplemented with 10 ng/mL macrophage CSF (PeproTech, 300−25) and 10% FBS (Gibco, 10099141) to generate Mφ.

### 4.5 Generation of human CAR‐T cells and tumor cell killing assay

Primary CD3 positive T cells were isolated from healthy donor PBMC via Dynabeads Untouched Human T Cells Kit (Thermo Fisher Scientific, 11344D) according to the instruction, and then activated with anti‐CD3 (5 μg/mL) and anti‐CD28 (5 μg/mL) antibodies for 2 days. The pre‐activated T cells were transfected with a second‐generation CAR‐T virus targeting NY‐ESO‐1, which was generated by GENECHEM. One day later, the CAR‐T cells were persistently activated with anti‐CD3/CD28 antibodies for about 2 days and then expended with 20 ng/mL IL‐2 for 5 days. SKOV3 tumor cells were pretreated with or without 10 ng/mL IL‐1β for about 48 h and then co‐cultured with those expanded CAR‐T cells at a ratio of 1:2 for 5 h, followed by determining the apoptosis of tumor cells with annexin V and propidium iodide staining. The cytotoxicity of CAR‐T cells was evaluated by granzyme B and IFN‐γ expression via flow cytometry. For the assessment of the cytotoxicity of CAR‐T cells affected by anti‐PD‐L1 antibody, the co‐cultured media were administrated with 10 μg/mL anti‐PD‐L1 antibody.

### 4.6 Cell stimulation

#### 4.6.1 Treatment of TCM

The SKOV3 cell line was seeded in six‐well culture plates (1 × 10^5^ cells per well) and cultured with 2 mL fresh McCoy's 5A (modified) medium. A549, U251, and U87MG cell lines were seeded in six‐well culture plates (1 × 10^5^ cells per well) and cultured with 2 mL fresh DMEM medium. The tumor culture media were collected after 48 h. The pre‐centrifuged tumor culture media were mixed with fresh RPMI 1640 medium at different ratios of 1:7, 1:3, and 1:1. THP1 Mφ was cultured with the mixed media for about 8 h for real‐time PCR to determine the transcriptional level of *Il‐1β* or 72 h for enzyme linked immunosorbent assay (ELISA) to detect secretion of IL‐1β in the supernatant.

THP1 Mφ was stimulated with the mixed media at a ratio of 1:1 about 8 h for real‐time PCR or 72 h for ELISA in the presence or absence of LARA (Selleck, S5400), AhRi (Selleck, CH223191), A2AR antagonist1 (Selleck, S8575), or mGluR5 antagonist (Selleck, S2645). THP1 Mφ was collected and lysed with TRIzol reagent (Sigma‐Aldrich, T9424). To detect secreted IL‐1β protein, the medium was centrifuged (800 g, 5 min) and the supernatant was collected. All samples were stored at −80°C for further use.

#### 4.6.2 Treatment of activated THP1 Mφ supernatant

Aiming to verify whether L‐lactate could trigger the THP1 Mφ to release IL‐1β following to potentiate the expression of CCL2 from tumor cells, the activated THP1 Mφ supernatant was collected following the stimulation with sodium L‐lactate (Sigma‐Aldrich, 71718) for 96 h. Untreated THP1 Mφ supernatant collected following 96 h of culture was used as a control. Activated THP1 Mφ supernatant was mixed with fresh McCoy's 5A (modified) medium or DMEM at a ratio of 1:1 and then incubated for about 8 h for real‐time PCR or 72 h for ELISA with tumor cells (1 × 10^5^ per well) in the presence or absence of IL‐1 receptor antagonist (IL‐1RA) (MedChemExpress, HY‐P7029). Tumor cells were then lysed using a TRIzol reagent. The concentration of secreted CCL2 protein was measured following the centrifugation of the final supernatant. All samples were stored at −80°C until further use.

#### 4.6.3 Treatment with IL‐1β

A2780, OVCAR8, SKOV3, A549, U251, and U87MG cell lines were seeded in six‐well culture plates (1 × 10^5^ cells per well). Recombinant human IL‐1β protein (PeproTech, 200−01B) was added to the wells at a final concentration of 10 ng/mL to stimulate the tumor cells.

### 4.7 Flow cytometry

Tumor cells were washed with PBS and stained with phycoerythrin (PE)‐conjuncted PD‐L1 antibody (29E.2A3, Biolegend) and then determined with flow cytometry (Beckman Coulter). Data were analyzed using Flow Jo software (Tree Star).

### 4.8 Reverse transcription‐PCR (real‐time PCR)

TRIzol reagent (Sigma‐Aldrich, T9424) was used to extract total RNA from cells. cDNA was synthesized from 1 μg of RNA using a Reverse Transcription Kit (Vazyme, R323‐01) following the manufacturer's protocol. Real‐time PCR was performed using the SYBR Green (Bio‐Rad Laboratories, 1725124) on a Bio‐Rad CFX Connect instrument. The quantification of the results was determined by the comparative Ct (2^−‐ΔΔCt^) method. The Ct value for each sample was normalized to the value of the glyceraldehyde‐3‐phosphate dehydrogenase (*GAPDH)* gene. Primer sequences for the *GAPDH*, *CD274*, *IL‐1β*, *CCL2*, and *NY‐ESO‐1* genes are as follows:

*GAPDH*, 5′‐GCATCCTGGGCTACACTGAG‐3′ (forward) and 5′‐AAGTGGTCGTTGAGGGCAAT‐3′ (reverse);
*CD274*, 5′‐CTGTCACGGTTCCCAAGGAC‐3′ (forward) and 5′‐GGTCTTCCTCTCCATGCACAA‐3′ (reverse);
*IL‐1β*, 5′‐CCACAGACCTTCCAGGAGAATG‐3′ (forward) and 5′‐GTGCAGTTCAGTGATCGTACAGG‐3′ (reverse);
*CCL2*, 5′‐AGAATCACCAGCAGCAAGTGTCC‐3′ (forward) and 5′‐TCCTGAACCCACTTCTGCTTGG‐3′ (reverse).
*NY‐ESO‐1*, 5′‐CTGAAGGAGTTCACTGTGTCCG‐3′ (forward) and 5′‐GCAGAAAGCACTGCGTGATCCA‐3′ (reverse).

All the primers were purchased from Qingke Biologics, Inc.

### 4.9 ELISA quantitation of IL‐1β and CCL2

To investigate which tumor‐derived stimulator triggers IL‐1β release from Mφ, the fresh TCM or boiled TCM or lactate was used to treat PBMC Mφ or THP1 Mφ for 72 h and then determined the IL‐1β level via a human IL‐1β ELISA Kit (DAKEWE, 1110122) according to the manufacturer's instructions.

To explore the CCL2 secretion, tumor cells were administrated with lactate‐primed THP1 Mφ culture medium or recombinant IL‐1β cytokine (10 ng/mL) for about 72 h, and then the CCL2 concentration in tumor supernatant was determined by a Human MCP‐1/CCL2 ELISA Kit (Biolegend, 438807) according to the manufacturer's instructions.

### 4.10 In vitro gene silencing

Silencing of IL‐1β in the tumor cell lines was achieved through in vitro small interfering RNA (siRNA) transfection according to the manufacturer's instructions, and the *IL‐1β* siRNA sequence was 3′‐CGATGCACCTGTACGATCA‐5′. The *IL‐1β* siRNA and negative control siRNA were purchased from Ribo Life Science. Tumor cells were seeded in six‐well plates (2 × 10^5^ cells/well) and transfected with 10 nM of control siRNA or IL‐1β siRNA. Silencing efficiency was assessed using real‐time PCR.

### 4.11 Immunohistochemistry

Ovarian cancer tissue samples were obtained with informed consent from 41 treatment naïve HG‐SOC patients at the Tongji Hospital of HUST, and the baseline information of these patients is applied in Table S1. The specimens were extracted during surgery, and tissues were formalin‐fixed and paraffin‐embedded. Standard IHC was performed using antibodies against granzyme B (Abcam, ab4059), CD68 (Abcam, ab125212), F4/80 (Servicebio, GB113373), PD‐L1 (Abcam, ab238697), and IL‐1β (Cell Signaling Technology, 12242S) using an Avidin‐Biotin Complex Vectastain Kit (Zsgb‐Bio, SP‐9000) according to the manufacturer's protocols. Tissue sections were scanned after immunostaining, and protein expressions were measured using Image J software. The diagnosis of high‐grade serous ovarian cancer (HG‐SOC) was confirmed by two independent pathologists.

### 4.12 Fluorescence multiplex immunohistochemical analysis

Fluorescence multiplex immunohistochemical analysis was performed according to the manufacturer's instruction. Briefly, paraffin‐embedded HG‐SOC patient tissues were cut into 4 μm consecutive sections, deparaffinized and antigen retrieved by a microwave oven for 15 min in EDTA buffer (pH 8.0). All the sections were incubated in four rounds of staining; in the order of CD8 (Abcam, ab237709), IL‐1β (Cell Signaling Technology, 12242S), granzyme B (Abcam, ab125212), and CD68 (Abcam, ab4059), each using a separate fluorescent tyramide signal amplification system: Quadruple‐Fluorescence immunohistochemical mouse/rabbit kit (Immunoway, RS0037). The information of HG‐SOC patients (*n* = 5) involved in the analysis is summarized in Table S1.

### 4.13 Immunofluorescence

Ovarian and lung cancer mouse tissue samples were obtained from the C57BL/6 ovarian and lung cancer model. The specimens were formalin‐fixed and paraffin‐embedded. Standard IF was performed with antibodies against IL‐1β (Cell Signaling Technology, 12242S), PD‐L1 (R&D SYSTEMS, AF1019), and CCL2 (ABclonal, A7277) according to the manufacturer's instructions. After immunostaining, the expression of IL‐1β, PD‐L1, and CCL2 was measured using Image J software.

### 4.14 Western blotting

Western blotting was performed according to standard methods. Primary antibodies included the following: GAPDH (Abcam, ab9485), p‐P65 (Cell Signaling Technology, 3033S), and PD‐L1 (Abcam, ab238697). SuperSignal West Pico Chemiluminescent Substrate kit (Pierce Biotechnology, Inc.) was used to determine the blots on a ChemiDoc XRS+ machine (Bio‐Rad Laboratories). BAY11‐7085 (Selleck, S7352) and SCH772984 (Selleck, S7101) were used for the pretreatment of cancer cells.

### 4.15 Tumor models

A volume of 100 μL PBS containing 1 × 10^7^ mouse ovarian cancer cells ID8‐VEGF^Hi^ was mixed with 100 μL Matrigel, and then 5 × 10^6^ cells (100 μL) within the mixture were injected subcutaneously into the right flank of the immune‐competent female C57BL/6 mouse. Similarly, 2 × 10^5^ mouse lung cancer cells LLC were transplanted subcutaneously to the right flank of the C57BL/6 mouse. Ten micrograms of neutralizing antibodies against mouse IL‐1β (BioXCell, BE0246) were administered intraperitoneally to each mouse twice a week from Day 0 (i.e., the day the tumor cells were administered), and 0.2 mg of neutralizing antibodies against PD‐1 (BioXCell, BP0273) were administered intraperitoneally to each mouse twice a week starting 7 days after the injection of tumor cells. Appropriate IgG controls (BioXCell, BP0085) were used. Tumor growth was measured every 3−4 days using an identical caliper by the same surveyor, and tumor volume was calculated as L × W × W × 0.5, where W was the width and L was the length.

### 4.16 Gene Expression Omnibus (GEO) datasets and The Cancer Genome Atlas (TCGA) cohort

Data on the correlation between IL‐1β and PD‐L1, IL‐1β and CCL2 expression in ovarian cancer (GSE12172), lung cancer (GSE19804), and glioma (GSE53733) were obtained from the Gene Expression Omnibus (1 http://www.ncbi.nlm.nih.gov/geo). The correlation analyses between IL‐1β and PD‐L1, IL‐1β, and CCL2 were performed via the R2 platform (2 https://hgserver1.amc.nl/cgi-bin/r2/main.cgi?open_page=login) and the GEPIA 2 platform (3 gepia2.cancer-pku.cn/#index). Survival analysis of the TCGA cohort was performed via the Kaplan–Meier plotter platform (4 http://kmplot.com/analysis/index.php?p=service&cancer=ovar).

### 4.17 Statistical analysis

GraphPad Prism 5 software was used to graph our data. Statistical significance was determined by unpaired two‐tailed Student's *t*‐test, one‐way analysis of variance (ANOVA), and linear regression analysis. A *p*‐value of less than 0.05 was regarded as significant.

## AUTHOR CONTRIBUTIONS

C.X. and Y.X. conceived the idea, performed the experiments, and wrote the manuscript. B.W.Z., E.K.D., H.Y.L., S.X., Z.W., S.Y.W., P.J., T.F., X.M.X., P.H., N.J., J.H.T., Q.Z., Y.X.C., Q.Z., and Y.F. participated in the experiments. J.H.T. and Y.F. were aware of the group allocation at the different stages of the experiment. F.Y. and Q.L.G. supervised the project. All authors read and approved the final manuscript.

## CONFLICT OF INTEREST STATEMENT

The authors declare no conflict of interest.

## ETHICS STATEMENT

All studies were approved by the Cancer Biology Research Center (Key Laboratory of the Ministry of Education) and the Ethics Committee of Tongji Medical College, Huazhong University of Science and Technology (Approval number: S248).

## Supporting information



Supporting Information

## Data Availability

All data are available from the corresponding authors upon request.

## Footnotes

1
http://www.ncbi.nlm.nih.gov/geo

2
https://hgserver1.amc.nl/cgi-bin/r2/main.cgi?open_page=login

3
gepia2.cancer-pku.cn/#index

4
http://kmplot.com/analysis/index.php?p=service&cancer=ovar
