# Supplementary material for: Macrophages facilitate tumor cell PD‐L1 expression via an IL‐1β‐centered loop to attenuate immune checkpoint blockade
Source: MedComm (2020). 2023 Mar 30;4(2):e242. doi: 10.1002/mco2.242 (PMC10063777; doi:10.1002/mco2.242)
Supplement: Supplementary file 1 — Supporting Information [file MCO2-4-e242-s001.docx]

**Macrophages facilitate tumor cell PD-L1 expression via an IL-1β-centered loop to attenuate immune checkpoint blockade**

Cheng Xu^1,2#^, Yu Xia^1,2#^, Bai-Wei Zhang^3#^, Emmanuel Kwateng Drokow^4^, Hua-Yi Li^1,2^, Sen Xu^1,2^, Zhen Wang^1,2^, Si-Yuan Wang^1,2^, Ping Jin^1,2^, Tian Fang^1,2^, Xiao-Ming Xiong^1,2^, Pu Huang^5^, Ning Jin^1,2^, Jia-Hong Tan^6^, Qing Zhong^1,2^, Yu-Xin Chen^1,2^, Qi Zhang^7^, Yong Fang^1,2^, Fei Ye^3*^ and Qing-Lei Gao^1,2*^

^1^ Department of Gynecological Oncology, Tongji Hospital, Tongji Medical College, Huazhong University of Science and Technology, Wuhan, China.

^2^ National Clinical Research Center for Obstetrics and Gynecology, Cancer Biology Research Center (Key Laboratory of the Ministry of Education), Tongji Hospital, Tongji Medical College, Huazhong University of Science and Technology, Wuhan, China.

^3^ Department of Neurosurgery, Tongji Hospital, Tongji Medical College, Huazhong University of Science and Technology, Wuhan, China.

^4^ Department of Radiation Oncology, Zhengzhou University People’s Hospital & Henan Provincial People’s Hospital, Zhengzhou, China.

^5^ Department of Obstetrics and Gynecology, The Second Affiliated Hospital, Wenzhou Medical University, Wenzhou, China.

^6^ Department of Obstetrics and Gynecology, The First People’s Hospital of Yunnan Province. The Affiliated Hospital of Kunming University of Science and Technology, Kunming, China.

^7^Department of Plastic and Cosmetic Surgery, Tongji Hospital, Tongji Medical College, Huazhong University of Science and Technology, Wuhan, China.

***Corresponding Authors:**

Qing-lei Gao, Cancer Biology Research Center (Key Laboratory of the Ministry of Education), Tongji Hospital, Tongji Medical College, Huazhong University of Science and Technology, 1095 Jiefang Anv. Wuhan, Hubei 430030, China. Tel: 00-86-83662681; Fax: 00-86-83662779; E-mail: [qingleigao@hotmail.com](mailto:qingleigao@hotmail.com).

Fei Ye, Department of Neurosurgery, Tongji Hospital, Tongji Medical College, Huazhong University of Science and Technology, 1095 Jiefang Anv. Wuhan, Hubei 430030, China. E-mail: yeyuanbei@hotmail.com.

^#^ These authors contributed equally to this work.

**Supplemental figures**


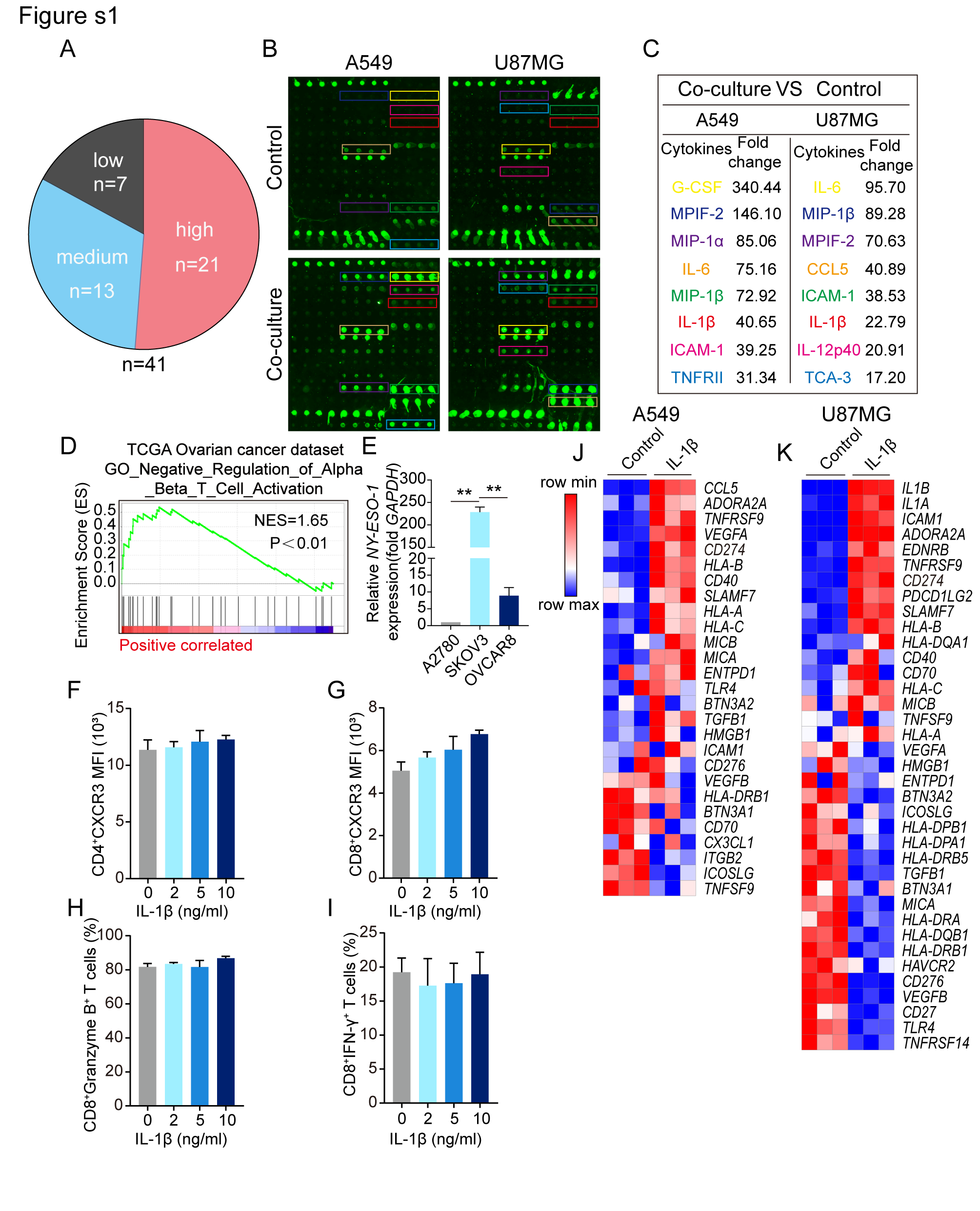


**Supplemental Figure 1 | IL-1β reprograms immune checkpoint signature genes expression in tumor cells without directly dampening T cell anti-tumor functions. A.** Pie chart of distribution of CD68^+^ Mφ ratio in ovarian cancer tissues (high＞15%, 5% ≤ medium ≤ 10%, low＜5%). **B-C.** Representative images of immune-related human inflammation array of THP1 Mφ and A549/U87MG co-culture medium. **D**. Gene set enrichment analysis (GSEA) plot of the association between gene sets that are positively correlated with IL-1β expression and a representative immunosuppression signature (n=594). **E.** Relative *NY-ESO-1* mRNA expression in SKOV3, OVCAR8, and A2780 was determined by real time-PCR (n=4). **F-I.** Primary human T cells were stimulated with anti-CD3/CD28 in the presence or absence of IL-1β at indicated concentrations for 3 days and then determined the expression of CXCR3, Granzyme B, and IFN-γ using flow cytometry. **J-K.** Heatmap showing the relative mRNA expression of the immune checkpoint signature genes in A549 (**J**) and U87MG (**K**).


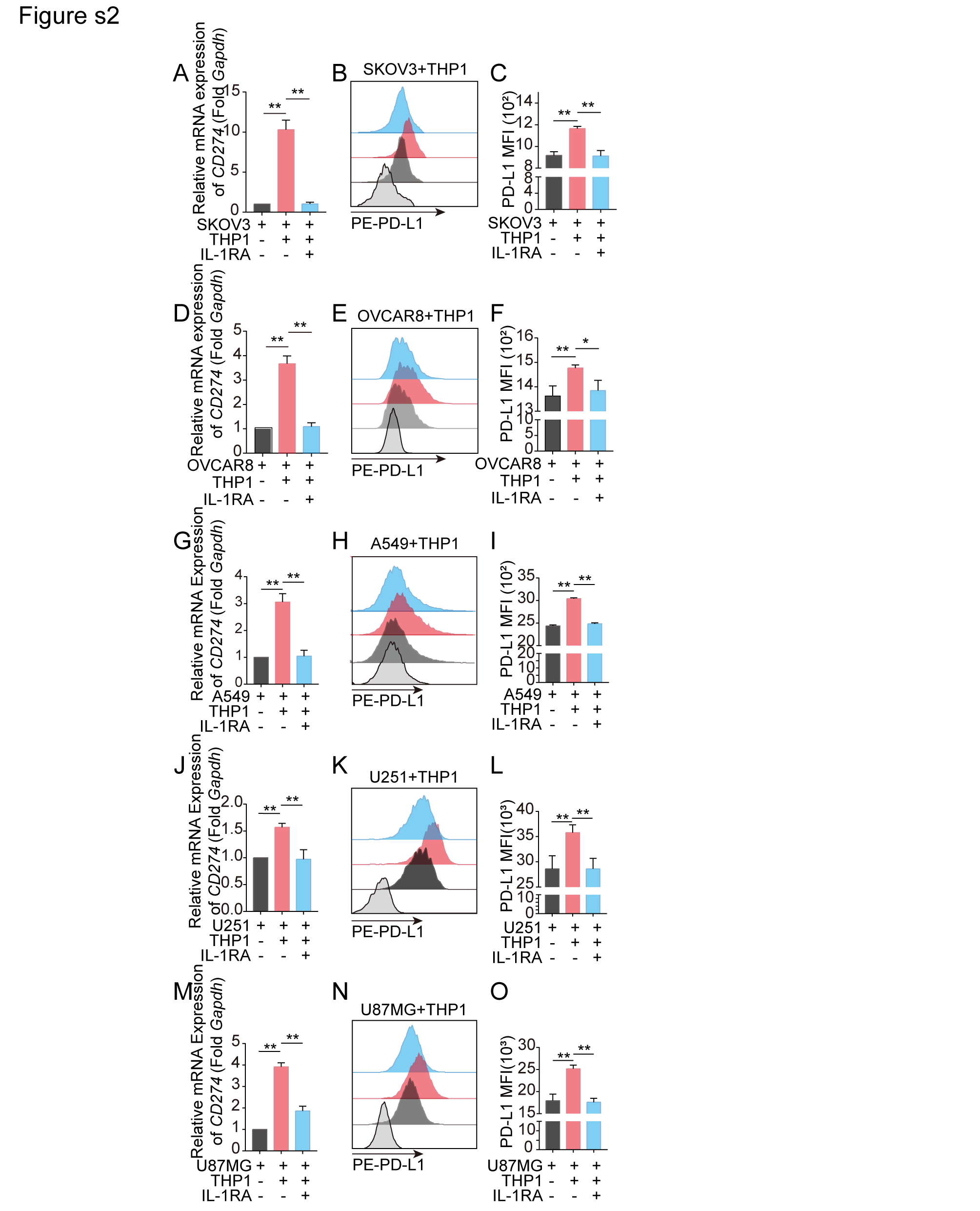


**Supplemental Figure 2 | PD-L1 expression of tumor cells is increased by IL-1β in the co-culture system. A, D, G, J, M.** The *CD274* mRNA expression was determined by real time-PCR in SKOV3, OVCAR8, A549, U251, and U87MG when co-cultured with THP1 Mφ in the presence or absence of IL-1RA pretreatment (48 h, n=4). **B-C, E-F, H-I, K-L, N-O.** Representative histograms and bar charts of MFI showing the membrane PD-L1 expression by SKOV3, OVCAR8, A549, U251, and U87MG after incubation with THP1 Mφ in the presence or absence of IL-1RA pretreatment for about 72 h (n=4). All graphs show mean ± SEM. Data were assessed by unpaired Student’s t-test. * *p* < 0.05, ** *p* < 0.01.


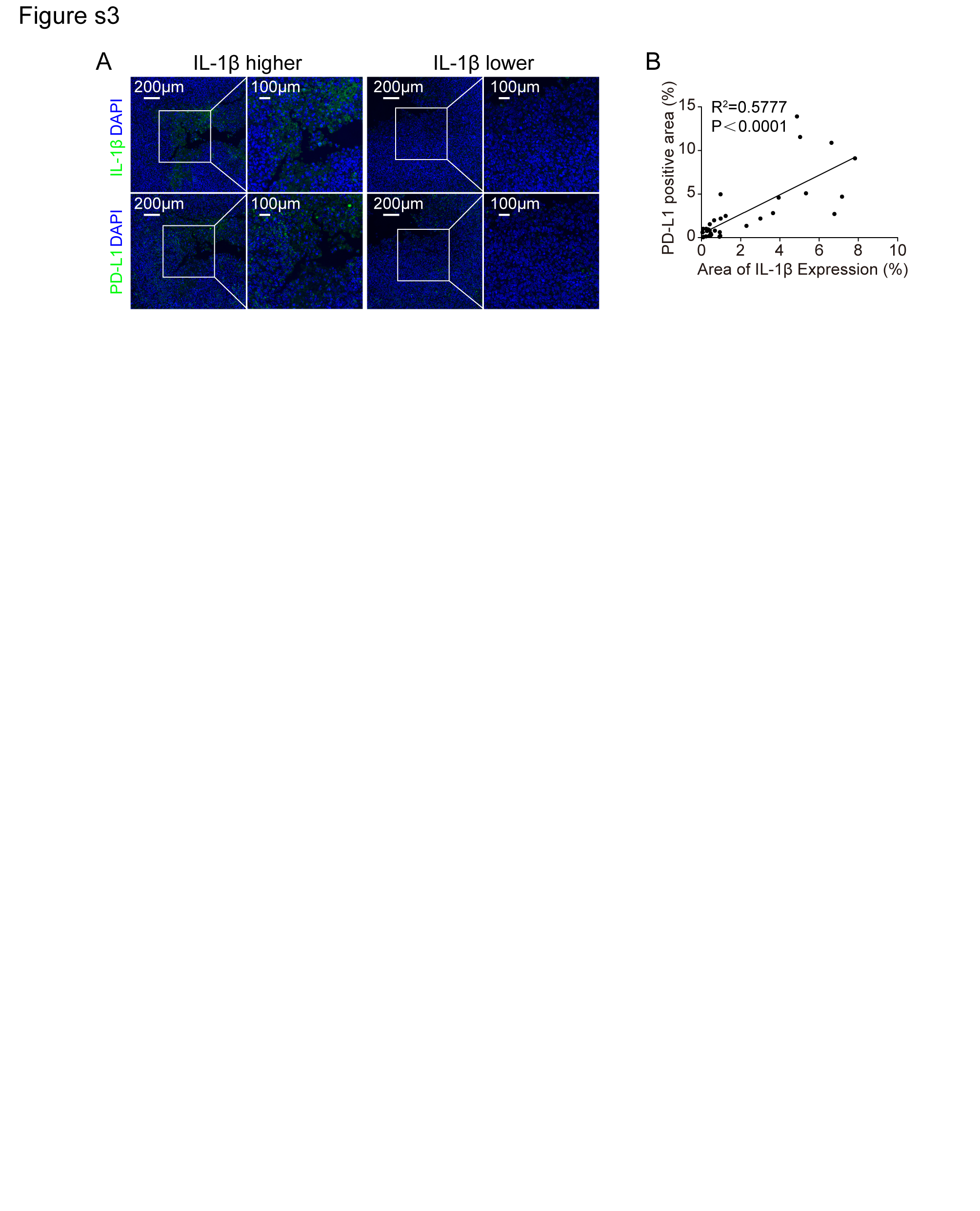


**Supplemental Figure 3 | PD-L1 expression of tumor cells in lung cancer mouse tissues is positively correlated with IL-1β secretion. A.** Representative images of immunofluorescence analysis of IL-1β secretion and PD-L1 expression in LLC lung cancer mouse tissues. **B.** Scatter plot of IL-1β secretion and PD-L1 expression levels in LLC lung cancer mouse tissues. Data were assessed using linear regression analysis, and 5 fields of each tumor section were selected randomly for calculating the ratios of positive area.


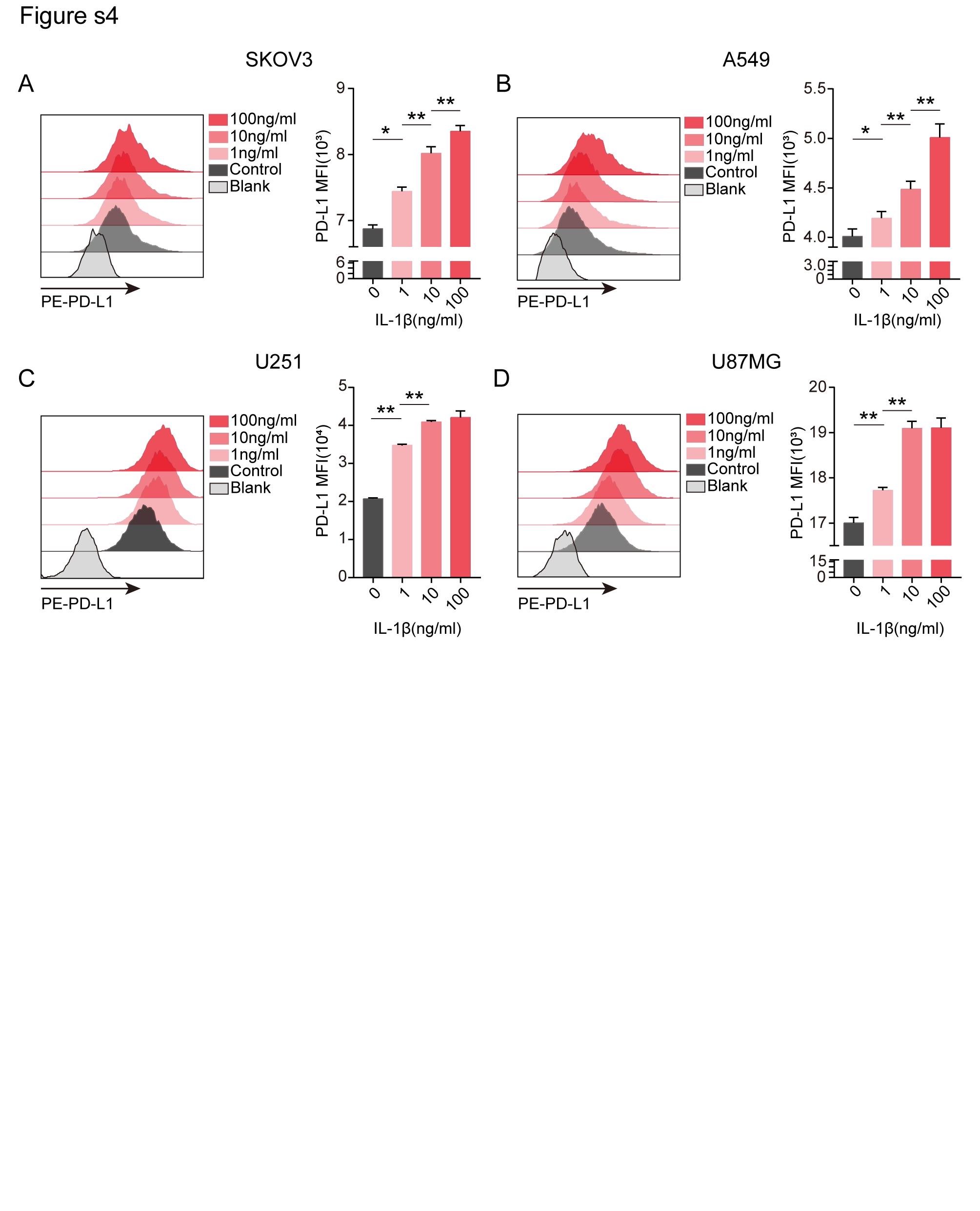


**Supplemental Figure 4 | IL-1β increases the expression of PD-L1 in a dose-dependent manner. A-D.** Representative histograms and bar charts of MFI showing the membrane PD-L1 expression of SKOV3, A549, U251, and U87MG after different concentrations of IL-1β treatment for 24 h (n=4). Data were assessed by unpaired Student’s t-test and represented as mean ± SEM. * *p* < 0.05; ** *p* < 0.01.


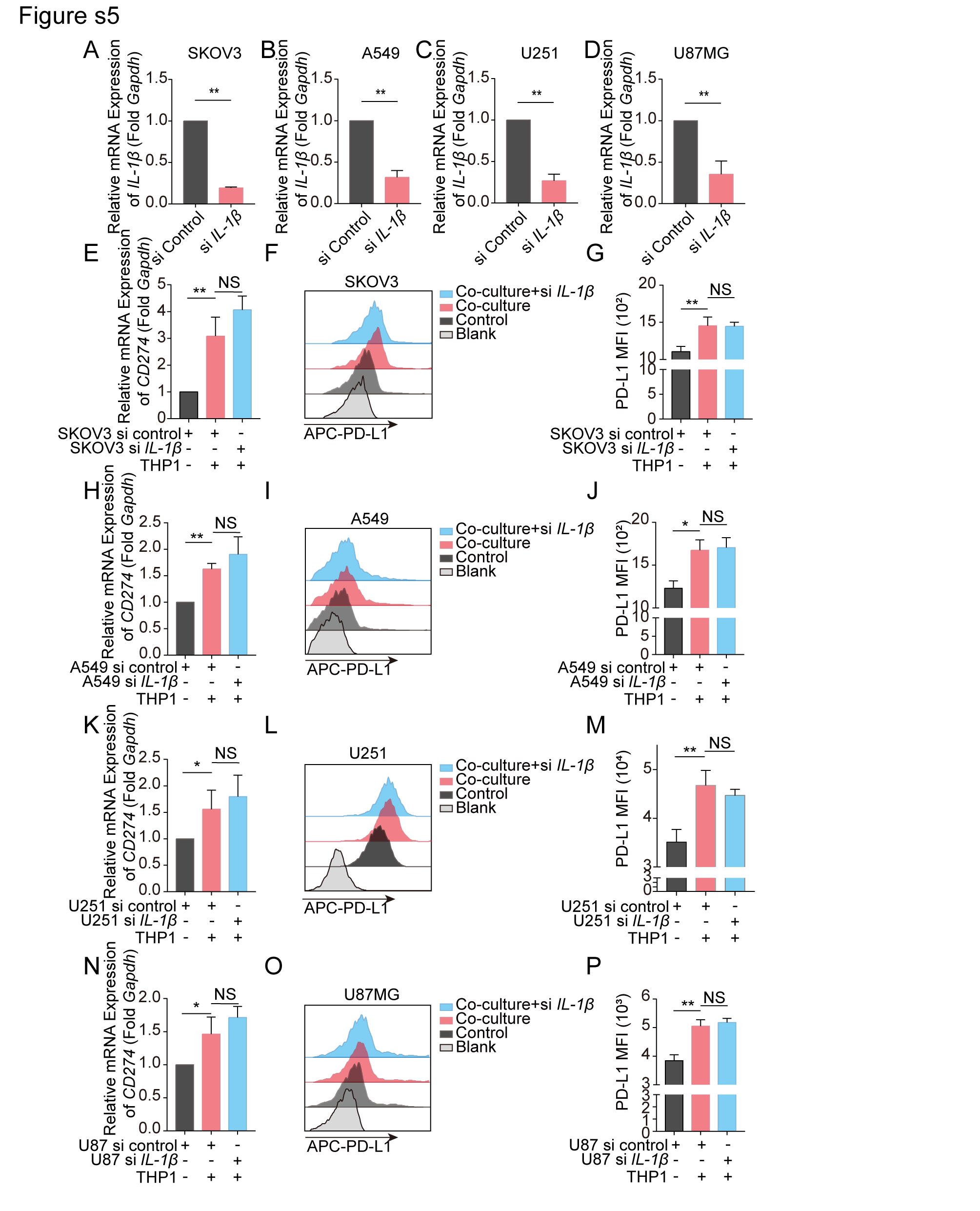


**Supplemental Figure 5 | Increased IL-1β is derived from Mφ rather than tumor cells in the co-culture system. A-D.** The *IL-1β* mRNA expression in SKOV3, A549, U251, and U87MG was determined by real time-PCR after these tumor cells were transfected with *IL-1β* siRNA (n=4). **E, H, K, N.** The *CD274* mRNA expression was determined by real-time PCR in tumor cells with or without *IL-1β* silencing when co-cultured with THP1 Mφ (48 h, n=4). **F-G, I-J, L-M, O-P.** Representative histograms and bar charts of MFI showing the membrane PD-L1 expression in tumor cells with or without *IL-1β* silencing when co-cultured with THP1 Mφ (72 h, n=4). All graphs show mean ± SEM. Data were assessed by unpaired Student’s t-test. * *p* < 0.05; ** *p* < 0.01. NS, no significance.


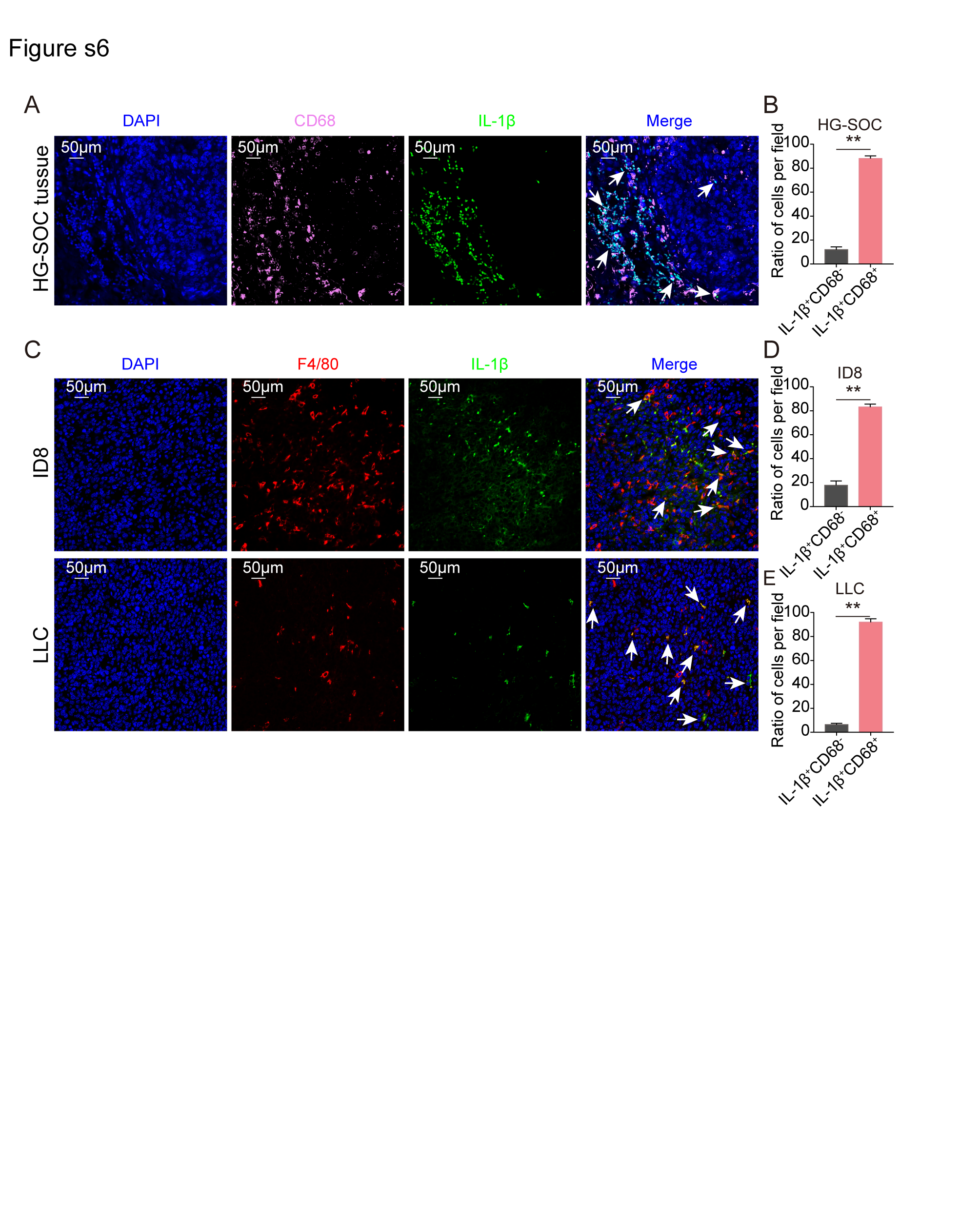


**Supplemental Figure 6 | Co-localization between IL-1β and Mφ in tumor tissues. A, C.** Representative images of Fluorescence multiplex immunohistochemical analysis (**A**) and immunofluorescence analysis (**C**) of the co-localization of IL-1β and CD68^+^ Mφ/F4/80^+^ Mφ in HG-SOC tissues and ID8/LLC mouse cancer tissues. **B, D, E.** Bar charts of the ratio of IL-1β and CD68^+^ Mφ/F4/80^+^ Mφ co-localization, 4 fields of each tumor section were selected randomly for calculating the ratio. Data were assessed by unpaired Student’s t-test. ** *p* < 0.01.


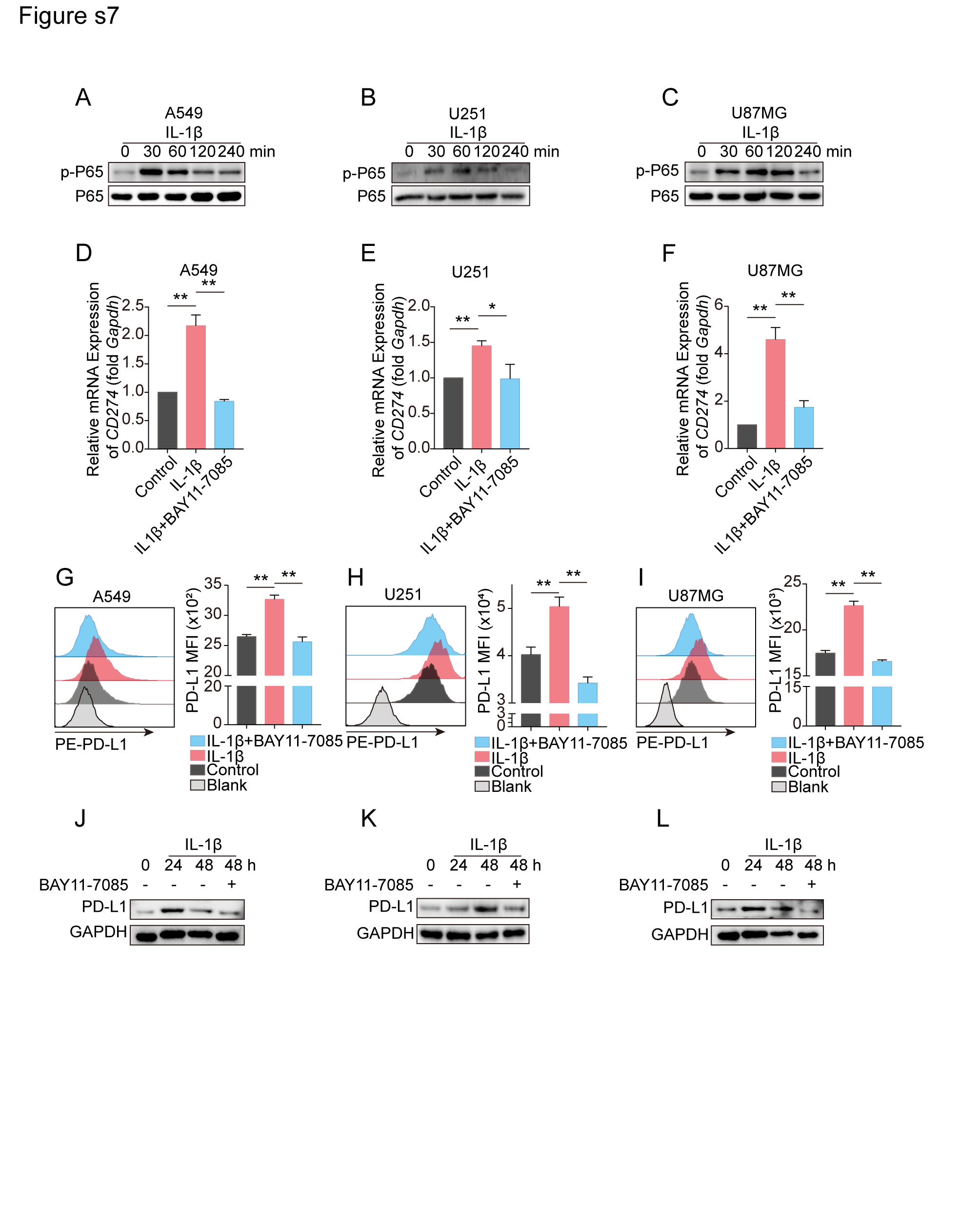


**Supplemental Figure 7 | IL-1β increases the expression of PD-L1 in lung cancer and glioma cell lines via the NF-κB signaling pathway. A-C.** A549, U251, and U87MG cell lines were treated with IL-1β (10 ng/mL) for indicated durations, and p-P65 were measured using western blotting. **D-F.** Tumor cells were treated with IL-1β (10 ng/mL) for 8 h in the presence or absence of pretreatment with BAY11-7085 (NF-κB inhibitor, 5 μM), n=4. The *CD274* mRNA expression was measured using real-time PCR (n=4). **G-I.** Representative histograms of MFI and bar charts showing membrane PD-L1 expression after IL-1β treatment with or without pretreatment with BAY11-7085 (NF-κB inhibitor, 5 μM), n=4. **J-L.** Tumor cells were treated with IL-1β (10 ng/mL) for indicated durations in the presence or absence of pretreatment with BAY11-7085 (NF-κB inhibitor, 5 μM). PD-L1 expression was measured using western blotting. All graphs show mean ± SEM. Data were assessed using an unpaired Student’s t-test. * *p* < 0.05; ** *p* < 0.01.


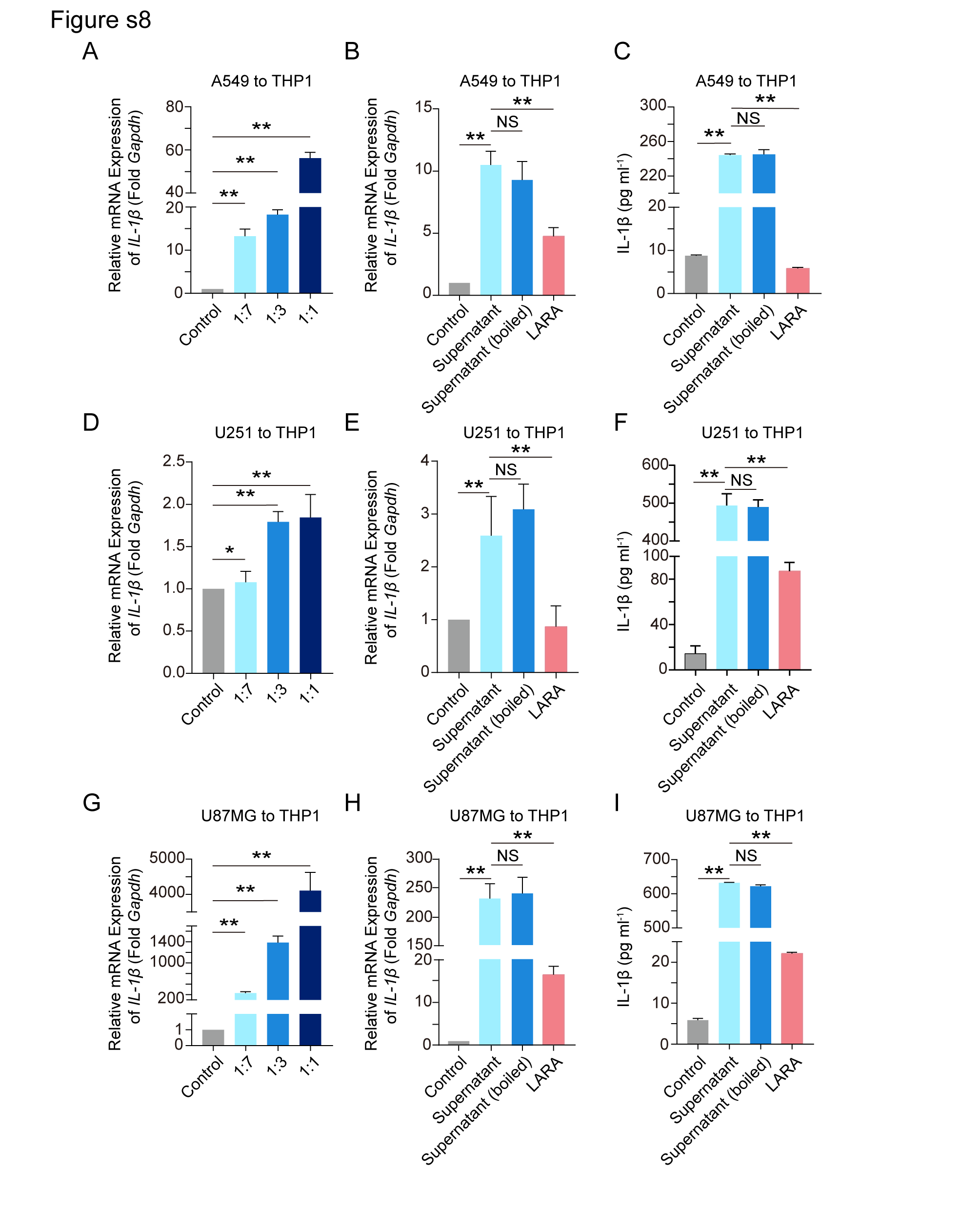


**Supplemental Figure 8 | Lactate secreted from lung cancer and glioma cells induces IL-1β transcription and secretion in THP1 Mφ. A, D, G.** A549/U251/U87MG supernatant mixed with fresh medium at the ratio of 1:1, 1:3, or 1:7 were used to treat THP1 Mφ for 2 h, and then the *IL-1β* mRNA expression in THP1 Mφ was determined using real-time PCR (n=4). **B-C, E-F, H-I.** THP1 Mφ was treated with A549/U251/U87MG supernatant or boiled supernatant (100 °C for 5 min) mixed with fresh medium in a 1:1 ratio in the presence (red) or absence of LARA for about 2 h to determine the IL-1β mRNA expression using real-time PCR (**B, E, H**) and for about 72 h to determine the IL-1β concentration in the medium by ELISA (**C, F, I**). All graphs show mean ± SEM. Data were assessed by unpaired Student’s t-test. * *p* < 0.05; ** *p* < 0.01. NS, no significance.


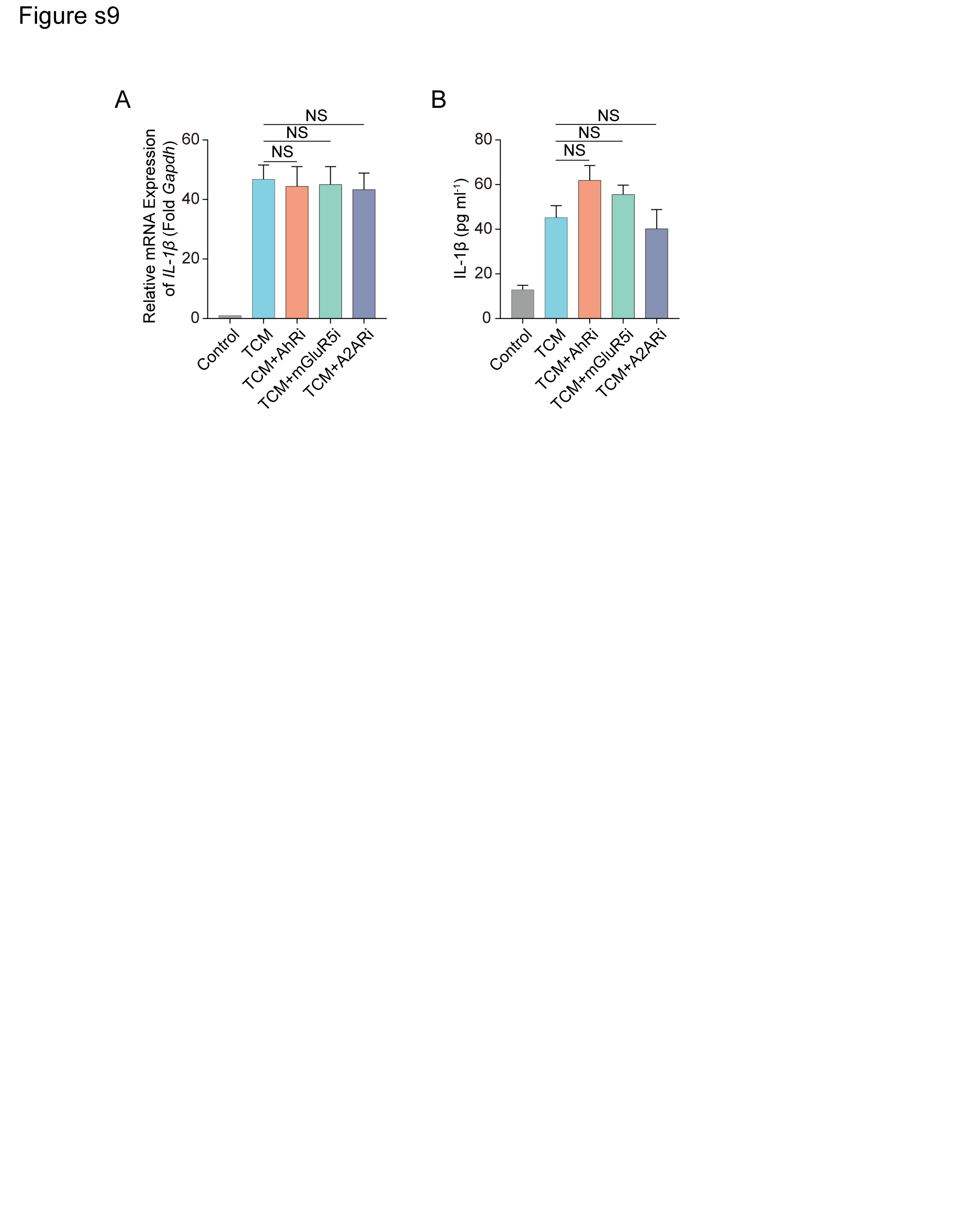


**Supplemental Figure 9 | AhR, mGluR5, and A2AR are not involved in the increased transcription and secretion of IL-1β in TCM. A-B.** THP1 Mφ was cultured in U87MG TCM with or without AhR, mGluR5, and A2AR inhibitors. The *IL-1β* mRNA expression in THP1 was determined by real-time PCR (8 h, n=4) (**A**). The secretion of IL-1β in the medium was determined by ELISA (96 h, n=4) (**B**). All graphs show mean ± SEM. Data were assessed by unpaired Student’s t-test. NS, no significance.


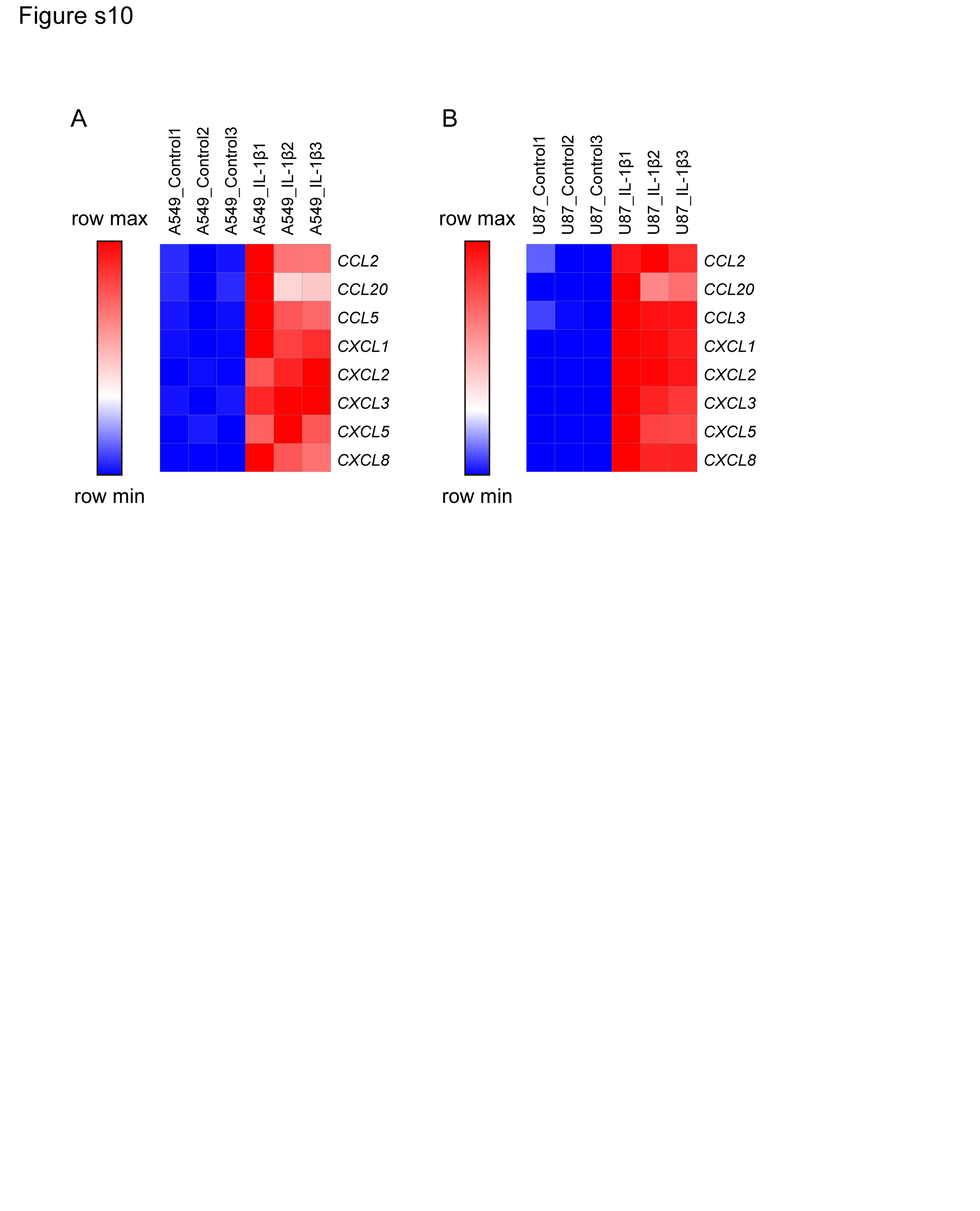


**Supplemental Figure 10 | IL-1β increases the transcription of cytokines including CCL2. A, B.** Heatmap showing the relative mRNA expression of the cytokine genes in A549 and U87MG.


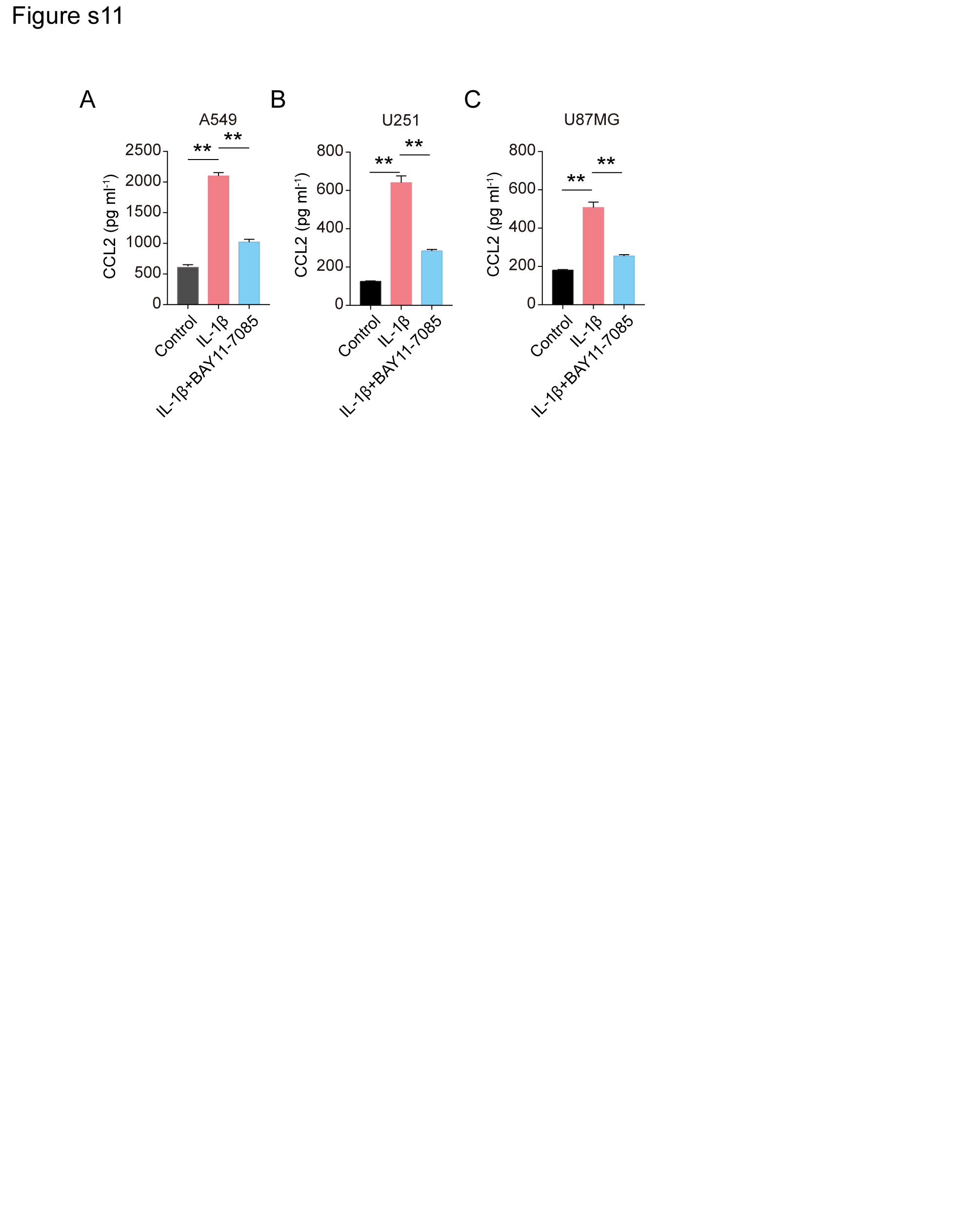


**Supplemental Figure 11 | IL-1β increases CCL2 secretion via activation of NF-κB signaling in lung cancer and glioma cells. A, B, C.** A549, U251, and U87MG cells were treated with IL-1β in the presence or absence of BAY11-7085 (NF-κB inhibitor, n=4) for about 72 h, and then the CCL2 secreted in the culture medium was determined by ELISA. All graphs show mean ± SEM. Data were assessed by unpaired Student’s t-test. ** *p* < 0.01.


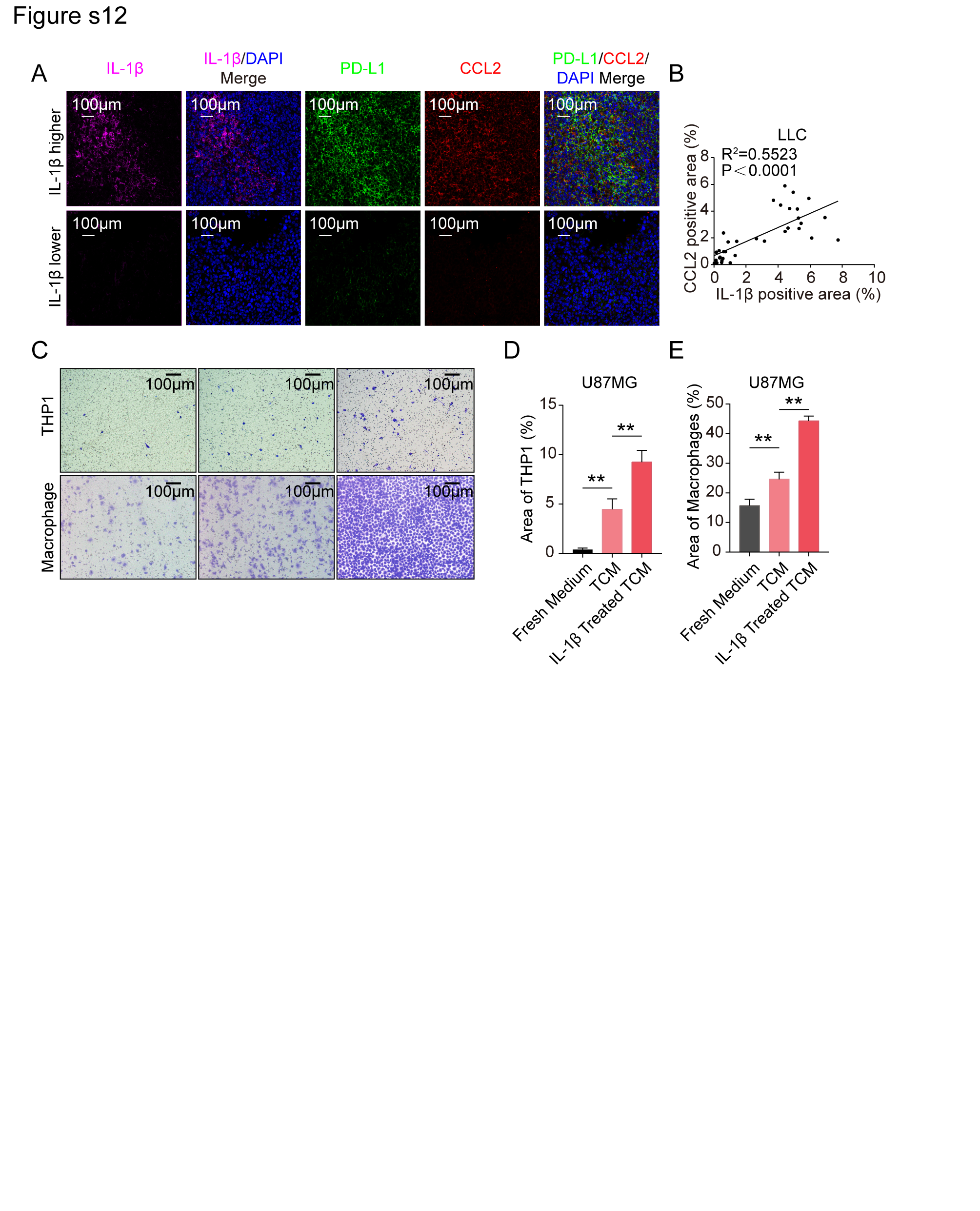


**Supplemental Figure 12 | IL-1β promotes CCL2 expression and Mφ infiltration. A.** Representative images of immunofluorescence staining with serial sections of lung cancer mouse tissues. IL-1β (purple) and DAPI were co-stained in one section (left two); PD-L1, CCL2, and DAPI were co-stained in another section (right three). **B.** Scatter plot of IL-1β and CCL2 secretion levels in lung cancer mouse tissues, and 5 fields of each tissue were selected randomly. **C.** Representative images of migration assays of THP1 Mφ and PBMC Mφ in fresh medium or TCM or IL-1β-activated TCM from U87MG. **D, E.** Bar charts of migration assays of THP1 Mφ (**D**) and PBMC Mφ (**E**) in fresh medium or TCM or IL-1β-activated TCM from U87MG, and 5 fields of each tissue were selected randomly. Data were assessed by unpaired Student’s t-test or linear regression analysis. ** *p* < 0.01.


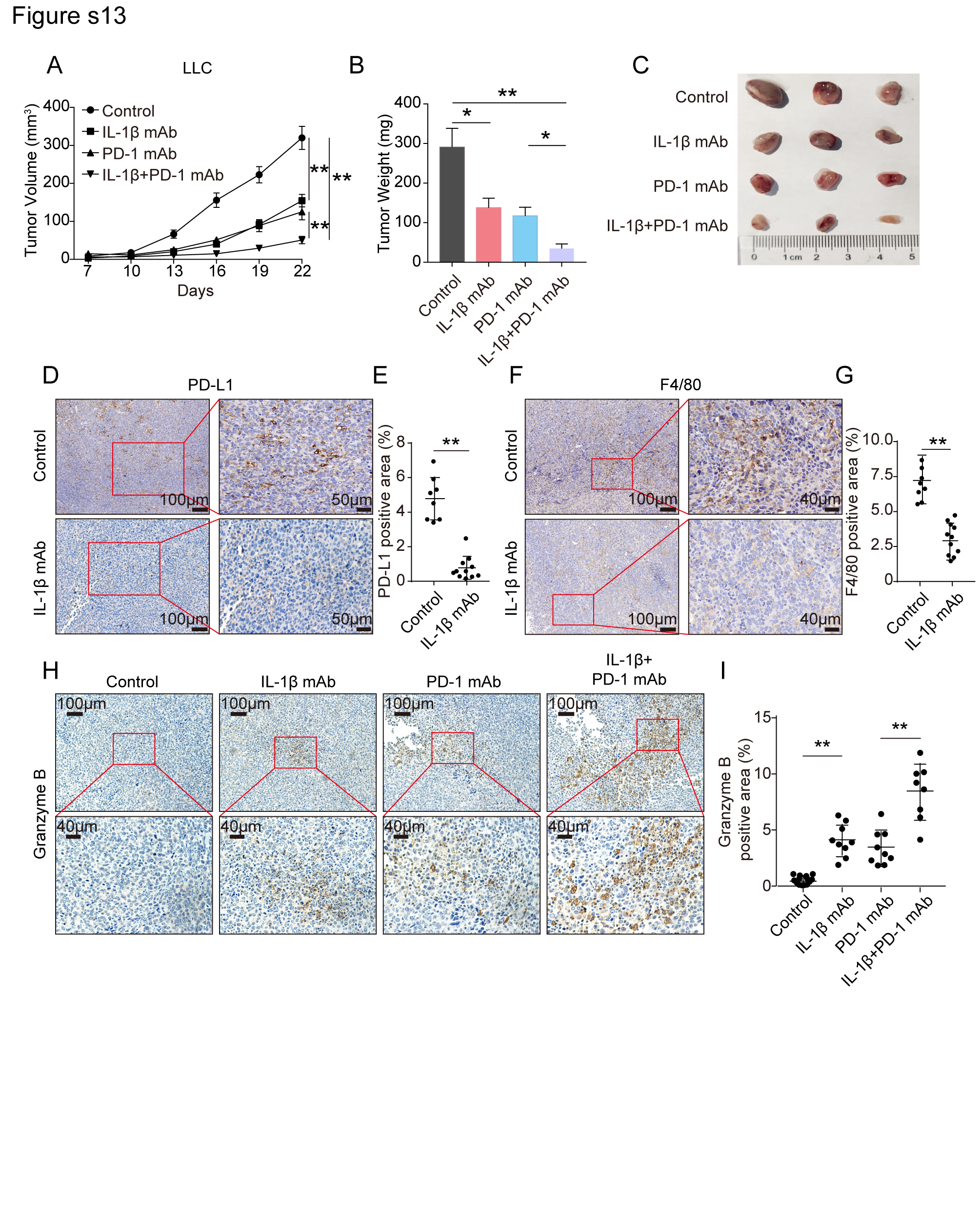


**Supplemental Figure 13 |** **Anti-IL-1β mAb exhibits anti-tumor effects mono-therapeutically or in combination with anti-PD-1 mAb in a LLC mouse model. A.** Tumor volume of control mice (●), mice treated with anti-IL-1β monoclonal antibody (mAb) (■), mice treated with anti-PD-1 mAb (▲), and mice treated with both anti-IL-1β and anti-PD-1 mAb (▼) in LLC lung cancer mouse models. **B.** Weights of LLC tumor tissues. **C. A** photo of LLC tumor tissues. **D.** Representative images of immunohistochemical analysis of PD-L1 in the lung cancer mouse model. **E.** Percentage of PD-L1 positive area in tumor sections from control and anti-IL-1β mAb groups, 2-3 fields of each tumor section were selected randomly for calculating the ratios of positive area. **F.** Representative images of IHC staining of F4/80^+^ Mφ in tumor sections from the lung cancer mouse model, 2-4 fields of each tumor section were selected randomly for calculating the ratios of positive area. **G.** Percentage of F4/80^+^ Mφ positive area in tumor sections from control and anti-IL-1β mAb groups. **H.** Representative images of IHC staining of granzyme B in mouse cancer sections from the lung cancer model. **I.** Percentage of the granzyme B positive area in tumor sections from the indicated treatment groups, 3-5 fields of each tumor section were selected randomly for calculating the ratios of positive area. All graphs show mean ± SEM. Data were assessed with unpaired Student’s t-test. * *p* < 0.05; ** *p* < 0.01.


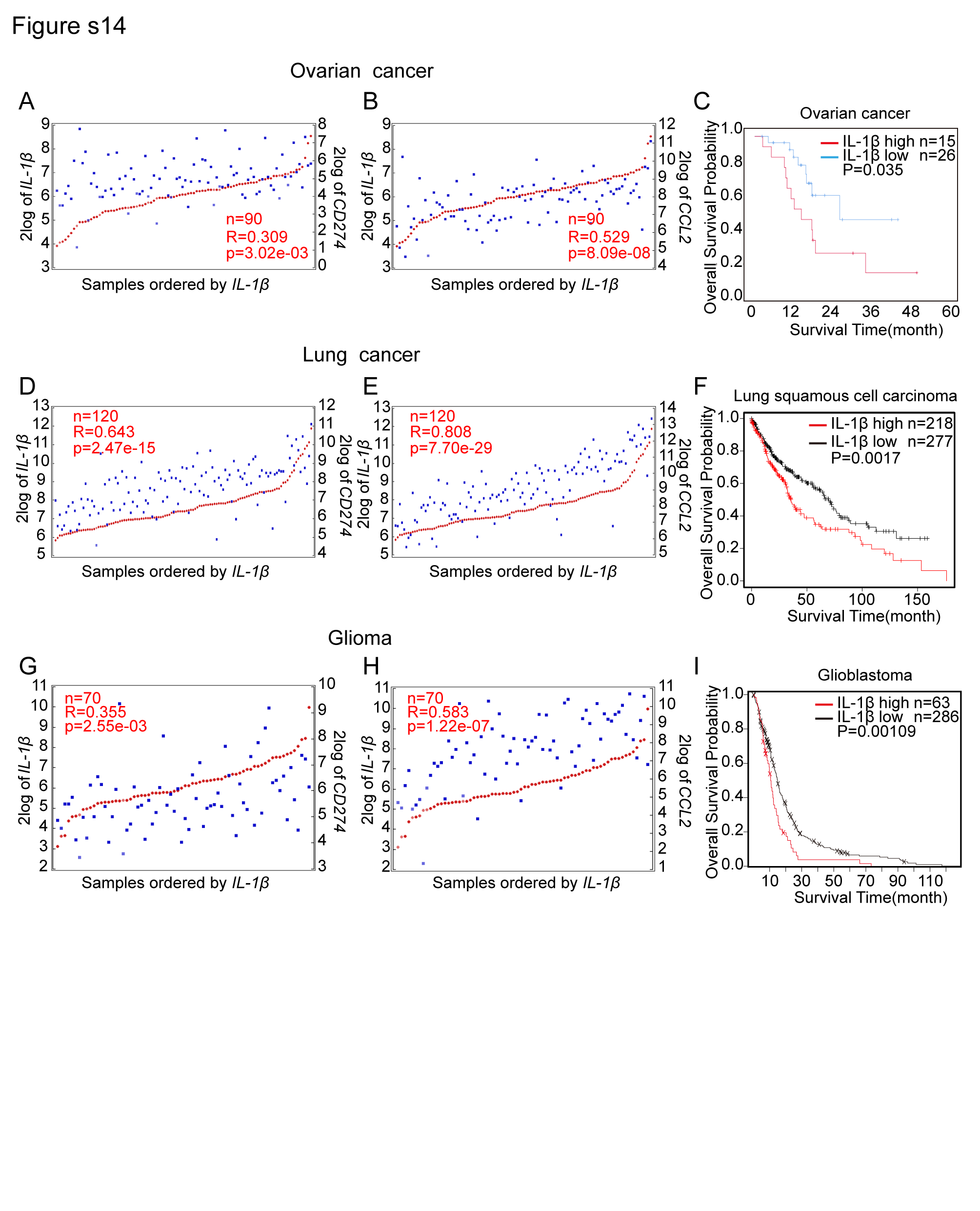


**Supplemental Figure 14 | PD-L1 and CCL2 expression in tumor cells are positively associated with IL-1β expression in the GEO cohort. A**, **D**, **G**. Correlation analysis of IL-1β and PD-L1 gene expression in ovarian cancer (n=90), lung cancer (n=120), and glioma (n=70) in GEO datasets. **B**, **E**, **H**. Correlation analysis of IL-1β and CCL2 gene expression in ovarian cancer (n=90), lung cancer (n=120), and glioma (n=70) in GEO datasets. **C**. Kaplan-Meier survival curves of 41 patients with ovarian cancer recruited at Tongji hospital, distinguished based on IL-1β secretion. **F, I**. Kaplan–Meier survival curves of lung squamous carcinoma (**F**) and glioblastoma (**I**) patients in TCGA cohort distinguished by IL-1β expression. All correlation analyses were processed via the R2 platform (https://hgserver1.amc.nl/cgi-bin/r2/main.cgi).


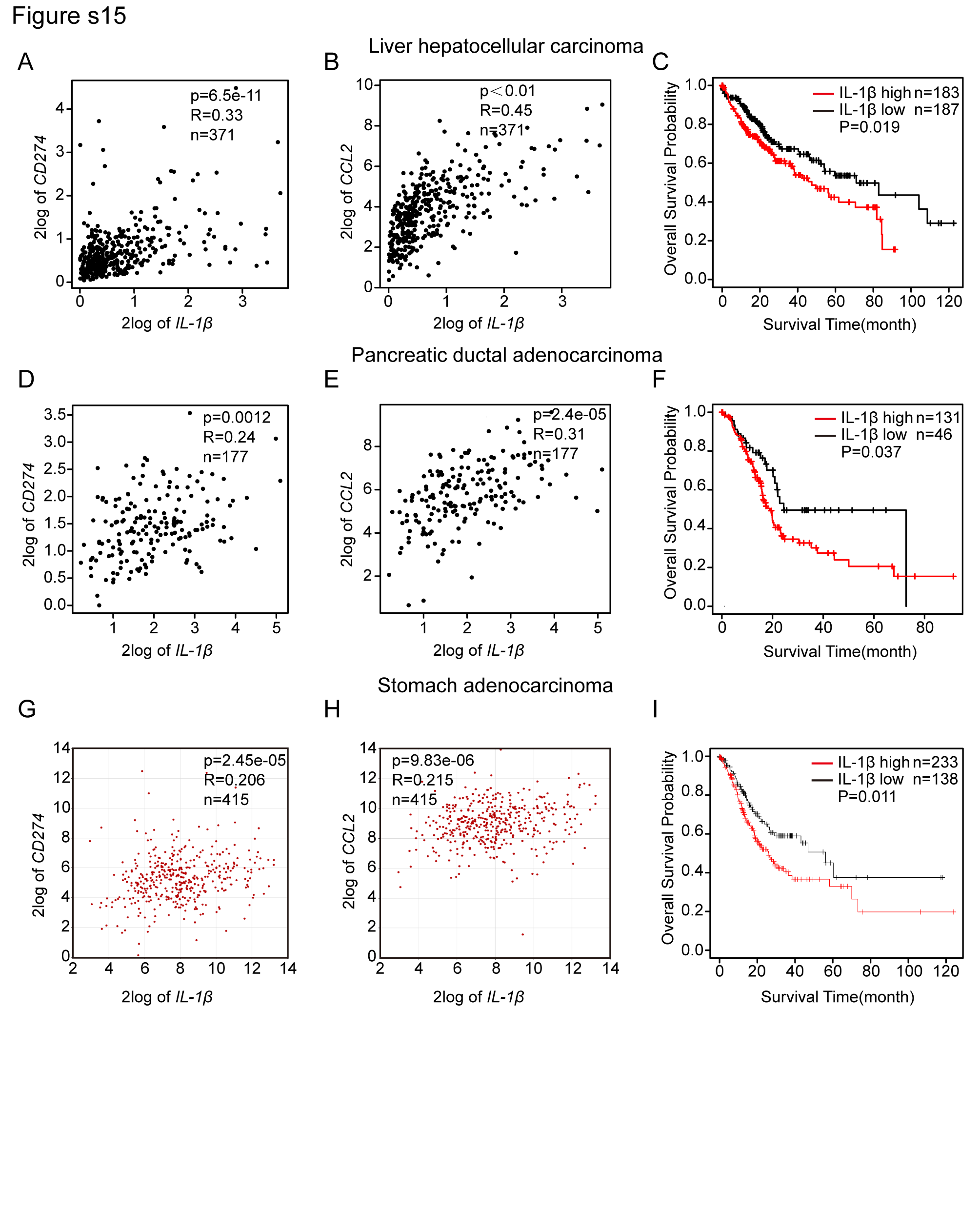


**Supplemental Figure 15 | The expression of PD-L1/CCL2 and the prognosis in the liver, pancreas, and stomach cancers are associated with IL-1β expression in the TCGA cohort. A, D, G.** Correlation analysis of IL-1β and PD-L1 genes expression in liver cancer (n=371), pancreas cancer (n=177), and stomach cancer (n=415) in the TCGA cohort. **B, E, H.** Correlation analysis of IL-1β and CCL2 genes expression in liver cancer (n=371), pancreas cancer (n=177), and stomach cancer (n=415) in the TCGA cohort. **C, F, I.** Kaplan–Meier survival curves of liver hepatocellular carcinoma, pancreatic ductal adenocarcinoma, and stomach adenocarcinoma patients in the TCGA cohort were distinguished by IL-1β expression. All correlation analyses were processed via the GEPIA 2 platform (<http://gepia2.cancer-pku.cn/#index>) and the R2 platform (<https://hgserver1.amc.nl/cgi-bin/r2/main.cgi>). All survival analyses were processed via the Kaplan-Meier Plotter platform (<http://kmplot.com/analysis/index.php?p=service&cancer=ovar>).


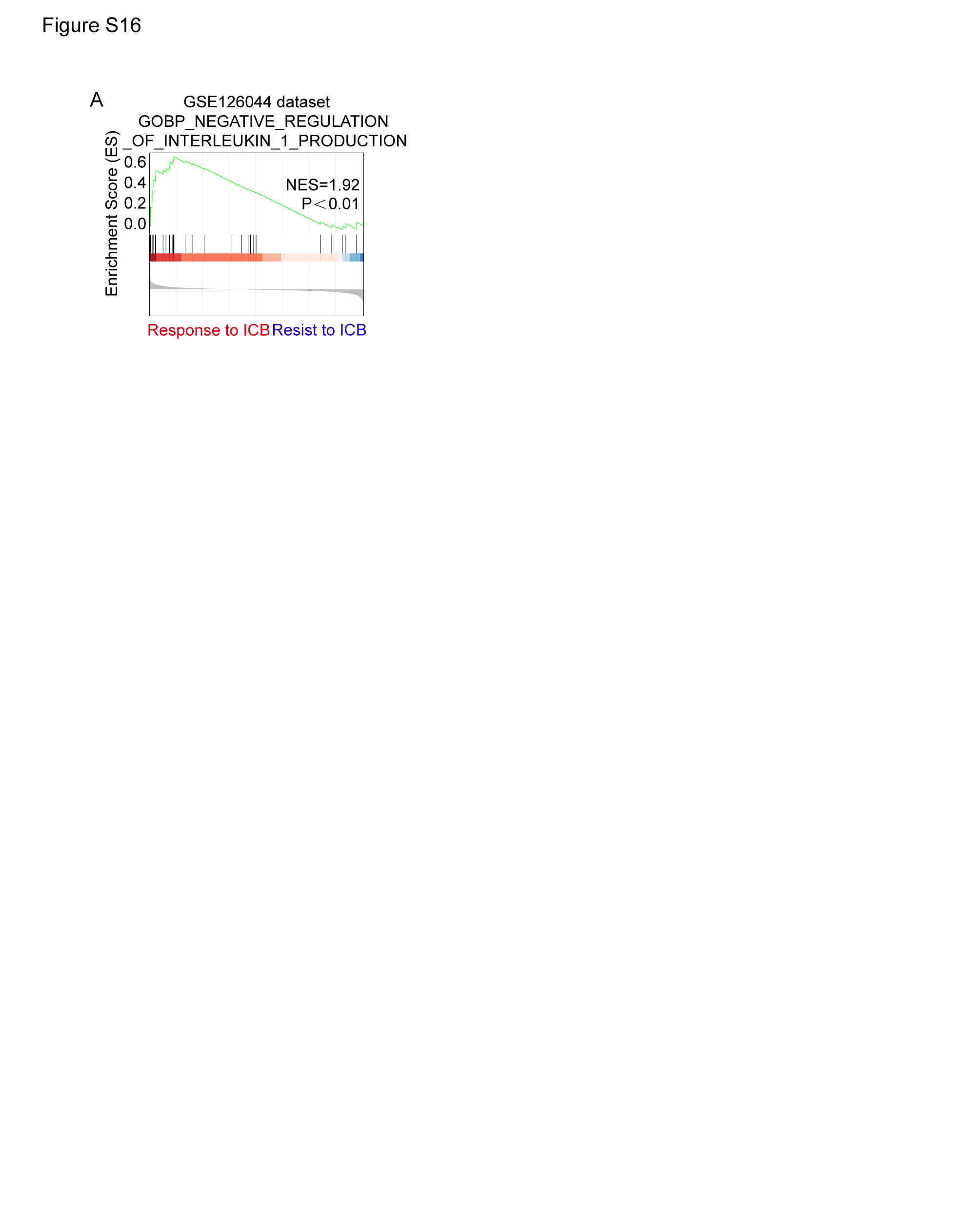


**Supplemental Figure 16 | Gene set enrichment analysis (GSEA) plot of the enrichment of negative regulation of IL-1β production in the ICB-responded lung cancer patients.**

**Supplementary Table 1. Characteristics and follow-up information of patients with ovarian cancer.**

| Patient No. | Age at diagnosis | Histology | FIGO stage | Debulking surgery | IL-1β expression | OS (days) | Status |
| --- | --- | --- | --- | --- | --- | --- | --- |
| 1 | 60 | HG-SOC | IIIc | yes | low | 3574 | alive |
| 2 | 52 | HG-SOC | IIIc | yes | low | 1335 | dead |
| 3 | 63 | HG-SOC | IIIc | yes | low | 1828 | alive |
| 4 | 52 | HG-SOC | IIIc | yes | low | 1222 | alive |
| 5 | 47 | HG-SOC | IIIc | yes | low | 914 | dead |
| 6 | 50 | HG-SOC | IIIc | yes | low | 194 | alive |
| 7 | 49 | HG-SOC | IIIc | yes | low | 1137 | dead |
| 8 | 50 | HG-SOC | UN | yes | low | 1519 | alive |
| 9 | 36 | HG-SOC | IIIc | yes | low | 1494 | dead |
| 10 | 55 | HG-SOC | IIc | yes | low | 3733 | alive |
| 11 | 16 | HG-SOC | IIIb | yes | low | 2289 | alive |
| 12 | 52 | HG-SOC | IIIc | yes | low | 1354 | dead |
| 13 | 54 | HG-SOC | IIIc | yes | low | 1412 | alive |
| 14 | 45 | HG-SOC | IIIb | yes | low | 357 | dead |
| 15 | 72 | HG-SOC | IIIc | yes | low | 1421 | alive |
| 16 | 49 | HG-SOC | IIIc | yes | low | 1021 | alive |
| 17 | 36 | HG-SOC | IIb | yes | low | 2219 | dead |
| 18 | 52 | HG-SOC | IIIc | yes | low | 1470 | alive |
| 19 | 68 | HG-SOC | IIIc | yes | low | 1500 | alive |
| 20 | 40 | HG-SOC | IIIc | yes | low | 492 | alive |
| 21 | 51 | HG-SOC | IIIb | yes | low | 1011 | dead |
| 22 | 48 | HG-SOC | IIIa | yes | low | 772 | alive |
| 23 | 50 | HG-SOC | IIc | yes | low | 1588 | alive |
| 24 | 44 | HG-SOC | IIb | yes | low | 914 | alive |
| 25 | 54 | HG-SOC | IIIc | yes | low | 1201 | alive |
| 26 | 69 | HG-SOC | IIIc | yes | low | 486 | alive |
| 27 | 61 | HG-SOC | IIIc | yes | high | 212 | dead |
| 28 | 51 | HG-SOC | IIIc | yes | high | 814 | dead |
| 29 | 65 | HG-SOC | IIIc | yes | high | 1585 | dead |
| 30 | 50 | HG-SOC | IIIc | yes | high | 1508 | dead |
| 31 | 62 | HG-SOC | IIIc | yes | high | 1033 | dead |
| 32 | 48 | HG-SOC | IIIc | yes | high | 777 | dead |
| 33 | 51 | HG-SOC | IIc | yes | high | 2893 | dead |
| 34 | 57 | HG-SOC | III | yes | high | 1219 | dead |
| 35 | 61 | HG-SOC | IIIb | yes | high | 846 | dead |
| 36 | 51 | HG-SOC | IIIc | yes | high | 1491 | dead |
| 37 | 58 | HG-SOC | III | yes | high | 435 | dead |
| 38 | 58 | HG-SOC | IIIb | yes | high | 4229 | alive |
| 39 | 49 | HG-SOC | IIb | yes | high | 2565 | alive |
| 40 | 57 | HG-SOC | IIc | yes | high | 951 | dead |
| 41 | 53 | HG-SOC | IIIa | yes | high | 1525 | alive |

Abbreviation: FIGO, International Federation of Gynecology and Obstetrics; HG-SOC, High-grade serous ovarian cancer; OS, overall survival.
